# Chimeric vaccine based on Iraqi HLA alleles against a predominant local *Escherichia coli* phylogroup

**DOI:** 10.3389/fimmu.2026.1875830

**Published:** 2026-08-18

**Authors:** Aziz Tamo Koro, Hiyam Adil Altaii

**Affiliations:** Department of Biology, College of Science, University of Mosul, Mosul, Iraq

**Keywords:** *Escherichia coli* pathotypes, faecal microbiota transplantation, HLA polymorphism, multi-epitope vaccine, UTI

## Abstract

**Introduction:**

*Escherichia coli* remains amongst the most globally important pathogens implicated in severe clinical manifestations. The progressive rise in multidrug-resistant strains highlights the urgent need for new vaccines. Therefore, this study was designed to develop a new multi-epitope vaccine containing the most conserved epitopes across *E. coli* pathotypes. Consequently, the study aimed to investigate the immunoadjuvant role of faecal microbiota transplantation in enhancing vaccine efficacy.

**Methods:**

Eighteen of the most conserved B-cell and T-cell epitopes of FimH, LptD, and BamA proteins were selected and included in a single construct. During the epitope selection process, HLA alleles predominant in the Iraqi population, as reported in previous studies, were used as criteria for selecting T-cell epitopes. The chimeric protein was expressed in BL21 *E. coli* and purified using affinity chromatography. Vaccine cross-protective immunity and protection were tested in *in vivo* experiments. Different formulations were used in the experimental evaluation: three doses of 100 μg of purified chimeric protein, injected intraperitoneally alone or encapsulated in PLGA nanoparticles, after faecal microbiota transplantation with and without gut microbiota modulation mediated by a cocktail of antibiotics. IgG1, IL-4, INF-γ, and NLRP3 levels were measured at 30 and 75 days after the first immunisation dose. Immunised mice were challenged with the local B2 UPEC phylogroup, and protection efficacy was considered 48 h later. Finally, the histological effects of the different chimeric protein formulations on the liver were assessed.

**Results:**

All vaccine formulations except those after faecal microbiota transplantation without gut microbiota modulation induce significant increases in IgG1, IL-4, and INF-γ levels at different times. Only vaccination after faecal microbiota transplantation with gut microbiota modulation elicited robust NLRP3 levels at 30 and 75 days after, and this was linked to the highest reduction in bladder bacterial load by 813-fold compared to the other formulations, as well as the mildest effect on liver histological changes.

**Discussion:**

These results demonstrated that the chimeric vaccine provides preliminary protection against a local B2 UPEC isolate. Furthermore, modulating gut microbiota via faecal transplantation markedly enhances the immunogenicity and protective efficacy of vaccination, suggesting its adjuvanticity.

## Introduction

*Escherichia coli* comprises several pathotypes that are critically implicated in many human clinical manifestations; amongst them, traveller’s diarrhoea, haemorrhagic colitis, haemolytic–uremic syndrome, inflammatory bowel disease, and dysentery are the most common intestinal diseases (1–4). On the other hand, urinary tract infection (UTI) is the most common extraintestinal infection caused by uropathogenic *E. coli* (5). Furthermore, *E. coli* pathotypes belong to many phylogroups, including A, B1, B2, D, E, F, and Clade I. In addition, all pathotypes are classified into one of these groups (6).

Globally, the emergence of multidrug-resistant (MDR) bacteria is a major health crisis. Accordingly, *E. coli* is amongst the pathogens that pose the greatest health risk worldwide (7–9), and a progressive rise in international MDR *E. coli* clones has escalated into a major public health concern (10). In Iraq, growing evidence documented a high prevalence of MDR *E. coli* amongst clinical isolates (11–13).

The alarming rise in multidrug resistance amongst *E. coli* pathotypes has prompted previous and recent studies to develop several promising vaccines against different strains. Traditional vaccines, such as live attenuated vaccines (LAVs) and killed vaccines, require culture, identification, selection of targeted components, validation of each component’s immunogenicity, and assessment of safety. In contrast, reverse vaccinology (RV), such as multi-epitope vaccines (MEVs), highly reduces the complexity and side effects of traditional vaccines (14).

To date, several candidate vaccines have been developed for *E. coli* pathotypes, including EPEC, EHEC, ETEC, and UPEC (6, 15). However, not one has yet achieved WHO approval. In this regard, several previous studies emphasise the use of multiple criteria to construct MEVs against *E. coli* pathotypes, such as UPEC, as the designed vaccine must account for strain heterogeneity and the virulence factors of different UPEC strains. Finally, UPEC vaccines should target one of the critical steps of pathogenesis (16).

Several surface epitopes have been exploited as major components of promising vaccines against UPEC; amongst them, FimH, a highly conserved fimbrial adhesin with an important role in the pathogenesis of UTI, is progressively used in MEVs (17–20). However, one of the important obstacles in developing UTI vaccines is the expansion of UPEC lineages, whilst the B2 phylogroup is well established as a primary *E. coli* strain causing UTIs; other phylogroups, such as A, B1, and E, that belong to InPEC, are also frequently implicated in UTIs (21, 22). It has previously been confirmed that different *E. coli* isolates from different geographical areas vary in distribution and antimicrobial resistance patterns (23); this highlights the need for future works to focus on local predominant *E. coli* phylogroups, as well as to utilise epitopes conserved by almost all *E. coli* pathotypes in designing new vaccines. This was analysed bioinformatically by Soltan et al. (24) who proposed that two main exposed proteins—lipopolysaccharide assembly protein (LptD) and outer membrane protein assembly factor (BamA)—are the most conserved proteins amongst *E. coli* pathotypes and could be perfect targets of a candidate vaccine.

Notably, inducing effective, long-lasting immune responses has remained a major challenge for previous MEVs. Although several earlier works utilised various types of adjuvants, such as Freund’s adjuvant (17), cholera toxin, and chitosan nanoparticles (20, 25), and despite the promising results, stimulating undesirable side effects remain an important drawback, highlighting the need to explore new-generation, safe, and inexpensive adjuvants.

Faecal microbiota transplantation (FMT) is a recent medical approach, primarily proposed as a treatment for recurrent *Clostridium difficile* infections in 2013 (26). Subsequently, several studies were carried out for the same objective (27, 28). Later, this approach was used in the United States as a first-line therapy for CDI (29–31) and in Europe (32). The FMT medical approach, characterised by several important benefits, is inexpensive, effective, safe, and first-line in the treatment of CDI (33). The underlying mechanism of FMT remains unclear; it is suggested that an increase in the abundance of some bacterial species is associated with positive outcomes via increased production of short-chain fatty acids (SCFAs), whilst other bacterial species are associated with negative outcomes. Some SCFA types, such as butyrate, play a crucial role in maintaining gut health and regulating immune responses. Later, studies clearly demonstrate that the transfer of probiotics via FMT is significantly associated with beneficial immune responses (34–37).

An inflammasome, a multiprotein oligomer, is a pattern recognition receptor (PRR). It plays a crucial role in activating innate immune responses and inflammatory reactions. It is also known as the NLRP3 inflammasome, a 115-kDa cytosolic receptor present in many immune and non-immune cells, such as neutrophils, dendritic cells, osteoblasts, lymphocytes, and epithelial cells (38). Recently, strong data propose a correlation between SCFA levels and NLRP3 expression (39, 40). This evidence clearly suggests a possible adjuvant role of SCFA in stimulating the NLRP3 inflammasome. Nevertheless, this correlation has not yet been studied in the context of vaccination and warrants further investigation.

In the present study, we aimed to develop a chimeric vaccine based on bioinformatic tools to select the most conserved epitopes of the FimH, LptD, and BamA proteins across *E. coli* pathotypes as potential vaccine components and evaluate its immunogenicity to protect against the most locally prevalent UPEC phylogroup as a model of *E. coli* infections. Furthermore, this study explores the use of FMT as a potent adjuvant platform to improve immune responses against targeted epitopes.

## Materials and methods

### Selection of target proteins

The VFDB bioinformatic tool was used to analyse and select the most appropriate *E. coli* proteins that encode for virulence factors. According to the analysis, three proteins—FimH, LptD, and BamA—were selected to be the targets for the construction of a candidate vaccine in the present study.

### Sequence retrieval and coverage

Protein sequences of FimH (access number P08191), LptD (access number P31554), and BamA (access number P0A940) were retrieved using the UniProt bioinformatic tool. The NCBI (BLASTp) was used to test the coverage of the sequences of targeted proteins with those of *E. coli* pathotypes.

### Selection of target protein sequences

Extracellular domains of the FimH, LptD, and BamA proteins were used as target sequences for the epitope prediction process in the present study. For this purpose, both PSORTb and TMHMM were used to predict extracellular domains (41).

### Epitope prediction

Two immunoinformatic tools, ABCpred and IEDB, were accessed to predict both T- and B-cell epitopes; only sequences overlapping between the two tools were further assessed as components of the candidate vaccine (42). Selection of T-cell epitopes is strictly limited to the most frequent HLA alleles in the Iraqi population reported in previous studies (43–46) and those reported in the Allele Frequency Net Database (https://www.allelefrequencies.net/hla.asp) as a basic criterion in the selection of MHC-I and MHC-II epitopes.

### Antigenicity, allergenicity, toxigenicity, and physicochemical evolution

#### Evolution of epitopes

The capability of overlapping predicted epitopes, as well as the final chimeric protein sequence, to be recognised by immune cells was predicted using the VaxiJen (version 2.0) bioinformatic tool. All epitopes above 0.4 were suspected antigens, and those below this value were excluded (47).

Allergenicity and toxigenicity of predicted epitopes and the final construct were tested using the AllerTOP (version 2.1) and ToxinPred online bioinformatic tools. All sequences were submitted and evaluated (48). The solubility was tested using the bioinformatic tool PepCalc from INNOVAGEN (https://www.innovagen.com/). Only epitopes that show good water solubility are selected and further evaluated.

### Prediction of INF-γ and IL-4 epitopes

Only those epitopes that stimulate INF-γ production as a cellular immune enhancer and IL-4 production as a humoral immune enhancer were selected in the present study; for this purpose, both IFN epitope and IL-4pred immunoinformatic tools were used.

### Conservation analysis of selected epitopes

Eighty FimH, 50 LptD, and 67 non-redundant protein sequences were retrieved from NCBI, followed by a multiple sequence alignment (MSA) using ClustalW/Omega online tool, and the conservativity percentage (C%) was calculated via the formula: C = $\frac{N\;\text{identical}}{N\;\text{total}}\times 100$, where *N* identical represents identical amino acid residues amongst sequences at a specific alignment, whilst *N* total rfers to the total number of aligned sequences. The WebLogo online server was used to generate a sequence logo to visualise amino acid frequencies calculated in bits.

### Final construction of the chimeric vaccine

Final MEV ordering was constructed manually, and the overall steps were as follows: methionine was inserted as the first amino acid to confirm accurate mRNA translation during gene expression. KK linker was used to link B-cell epitopes. GGGS linker was used to link Tc-cell epitopes. GPGPG linker was used to link Th-cell epitopes. EAKK linker was used to link internal adjuvants as well as between adjuvant and selected epitopes. Human beta-defensin sequence (NP_001075020.1 accession number in NCBI) was fused at the N-terminus of the final construct. Synthetic PADRE sequence (AKEVAAWTLKAAA) as THL adjuvant was incorporated as a second internal adjuvant. At the C-terminal of the final construct, a poly-histidine sequence was added to confirm a proper purification of the target protein.

### Characterisation of the final construct

Several characteristics, including molecular weight, number, composition, amino acid sequence, theoretical pI, instability index, and hydropathicity, were evaluated using the ProtParam bioinformatic tool.

### Secondary and tertiary structures of the final construct

Secondary structures, including helices, strands, and coils, of the final construct of the candidate vaccine were predicted using the PSIPRED online tool, whilst the AlphaFold3 online tool was used to predict the three-dimensional (3D) tertiary structure.

### Refinement and validation of the tertiary MEV structure

The 3D tertiary structure was constructed by the AlphaFold3 online server, refined using Galaxy Refine, and further validated by the stereochemical quality of the Ramachandran plot from PROCHECK, and the *Z*-score was calculated by ProSA-web.

### Molecular docking of the candidate vaccine

In the present study, the refined 3D structure was molecularly docked with TLR4 as follows: The final refined structure of the candidate vaccine was further edited in UCSF Chimera by adding both hydrogen atoms and charges. The 3D structure of TLR-4 retrieved from the PDB (accession number 3FXI) was also edited by removing all H_2_O molecules and non-structural amino acids. Docking between TLR-4 and the candidate chimeric protein was performed using the HDOCK online server. Hydrogen interactions between the chimeric vaccine and TLR-4 were assessed using the Protein-Ligand Interaction Profiler (PLIP) online tool.

### Codon optimisation

The JCat bioinformatic tool was used to convert the final constructed sequence into codon sequences to confirm optimal protein expression. The final nucleotide sequences were registered and deposited in the GenBank repository under accession PZ267928.

### Tandem repeats calculation

To avoid restriction enzyme-mediated cutting, the final construct was analysed with the Tandem Repeats Finder (TRF) bioinformatic tool to confirm the absence of tandem repeats.

### *In silico* cloning

*In silico* cloning was performed using the SnapGene software, and the final gene construct for the chimeric protein was mapped between the EcoR1 and HindIII restriction sites of the pET-30a plasmid.

### Expression and purification of the chimeric protein

The entire gene was synthesised by Macrogen (Korea) and subcloned into pET-30a+ at 4 µg. The expression vector was transformed into competent BL21 *E. coli* using a heat-shock protocol, then cultured on LB agar with kanamycin (10 µg/mL). A single positive clone was subsequently mixed with 10 mL of LB broth containing kanamycin (10 µg/mL). For protein expression, 10 mL of overnight growth was inoculated in LB broth with kanamycin (10 µg/mL), then induced by 1 mM IPTG (Sigma-Aldrich, USA) at the exponential phase (0.5 optical density at 600 nm), then incubated at 25 °C overnight. Protein was purified under native conditions using a Ni-NTA column (Cytiva, USA) and confirmed by sodium dodecyl sulphate–polyacrylamide gel electrophoresis (SDS-PAGE).

### PLGA encapsulation of the chimeric protein

To encapsulate the chimeric protein into PLGA nanoparticles (approved by the FDA as a promising novel delivery system), a water-in-oil-in-water (W1/O/W2) double-emulsion solvent-evaporation method was used as described by Unsunnidhal et al. (49).

### *In vivo* evaluation of the chimeric vaccine

#### Ethical consideration

Experimental protocols, including albino mice, were reviewed and approved by the Institutional Animal Care and Use Committee/University of Mosul/College of Veterinary Medicine (ref: UM.VET.2025.114 ON 15 July 2025).

### *In vivo* evolution

Fifty female albino mice (6–8 weeks) were used in the present study and housed in five clean separate cages provided with standard conditions (12 h light, 12 h dark, clean water, appropriate nutrition); all mice were observed for 1 month before vaccine challenge to eliminate unhealthy mice and divided equally into five groups: (1) Control group: intraperitoneally injected with PBS three times at 7, 14, and 30 days; (2) MEV group: intraperitoneally injected with 0.1 mL of 100 µg of the chimeric vaccine three times at 7, 14, and 30 days; (3) MEV+PLGA group: intraperitoneally injected with 0.1 mL of 100 µg of MEV + 100 µg of PLGA three times at 7, 14, and 30 days; (4) FMT+MEV group: Orally challenged with 0.1 mL of faecal slurry for three consecutive days, then the mice were intraperitoneally injected with 0.1 mL of 100 µg of MEV three times at 7, 14, and 30 days; and (5) A/F group: The antibiotic washout prior to the FMT (A/F) group was treated orally with 100 mg/kg of broad-spectrum antibiotics, ciprofloxacin and levofloxacin, twice daily for 3 days to wash out gut microbiota, subsequently challenged orally with 0.1 mL of faecal slurry for three consecutive days, then the mice were intraperitoneally injected with 0.1 mL of 100 µg of MEV three times at 7, 14, and 30 days. The FMT described in the protocol by Parker et al. (50) was used in the present study.

### Selection of the UPEC strain

A local B2 UPEC isolate from Iraqi patients with UTI was selected for an experimental bladder challenge. The selection process was based on phenotypic and phylogenetic analysis. Antibiotic resistance profiles were performed using the VITEK2 system and a described protocol by Clermont et al. (51) to analyse the phylogenetic background based on the presence/absence of four DNA markers (*chuA, yjaA*, TspE4.C2, and *arpA*). The selected isolate exhibits complete resistance to 15 different antibiotics, namely, Ampicillin, Piperacillin, Cefoxitin, Ceftazidime, Ceftolozane, Aztreonam, Doripenem, Tobramycin, Levofloxacin, Doxycycline, Minocycline, Tetracycline, Tigecycline, Chloramphenicol, and Colistin. Furthermore, phylogenetic analysis demonstrates the presence of *chuA*, *yjaA*, and TspE4.C2, and the absence of the *arpA* marker, reflecting the phylogenetic characteristics of the B2 phylogroup.

### Bladder challenging experiment

Eighty-two days following the first immunisation dose, four mice from each group were anaesthetised with a mixture of ketamine (70 mg/kg) and xylazine (5 mg/kg), and subsequently challenged transurethrally with 2 × 10^8^ CFU/mL of the local B2 UPEC isolate using a polyethene transurethral catheter. After 48 h of challenge, all mice were sacrificed and bladders were removed, homogenised with normal saline, diluted, and cultured onto Mueller-Hinton agar; the number of bacterial cells was calculated after 24 h of incubation at 37 °C.

### Measuring humoral immune response

All immunological parameters, including IgG1, IL-4, INF-γ, and NLRP3, were measured in serum at 30 and 75 days after the first immunisation dose of the chimeric protein by enzyme-linked immunosorbent assay (ELISA) using commercial kits (SUNLONG/China).

### Liver histopathological changes

Liver samples were firstly treated with different concentrations of ethyl alcohol (70%, 80%, 90%, 95%, and 100%) to dehydrate the samples, then the alcohol was removed during the cleaning step by submerging the samples in xylene. The samples were embedded in a special template and filled with pure molten paraffin wax. After the blocks were chilled on ice, the liver samples were sectioned into thin slices (approximately 5 µm) using a microtome, loaded into a water bath at 45 °C, and then placed on glass slides. All smears were treated with xylene to remove paraffin residues, then with ethyl alcohol to remove water. Subsequently, all samples were stained with haematoxylin for 10 min and washed four times with DW, followed by staining with alcohol eosin for 10 min and re-washing with DW. Finally, glass slides were treated with ethyl alcohol and xylene for final clarification, then coverslips were applied, and the slides were examined under a light microscope at 10× and 40× magnification.

### Statistical analysis

GraphPad Prism 8 was used for statistical analysis in the present study. All parametric results are visualised as mean ± standard deviation. Before statistical analysis, data normality was assessed using the Shapiro–Wilk test. Two-way analysis of variance (ANOVA) at *p*-value 0.05 followed by Tukey multiple comparison test was used to compare the concentrations of humoral immunological parameters (IgG1, IL-4, INF-γ, and NLRP3) at two different times, and one-way ANOVA at *p*-value 0.05 followed by Tukey multiple comparison test was used to compare bacterial reduction efficacy. Kruskal–Wallis test followed by Dunn’s multiple-comparison test with multiplicity adjustment was used to compare the histological changes.

## Results

### *In silico* design and structural analysis of the candidate chimeric vaccine

#### Selection of target proteins, sequence retrieval, conservativity, and extracellular domains of the selected proteins

Three proteins (FimH, LptD, and BamA) were selected as the main components of the chimeric vaccine. To confirm the pathogenicity role of the selected components, the VFDB bioinformatic tool was used. In the designing process, the candidate proteins were considered. FimH was selected owing to its crucial role in mediating UTI, supporting specifically with the utilisation of an experimental UTI model to stimulate *E. coli* infection. Meanwhile, the selection of both LptD and BamA proteins was stated by our study’s architectural objective to establish possible pan-pathotypes coverages against *E. coli*, and this rationale was strongly supported by the finding of Soltan et al. (24) suggesting broad-spectrum immunogenic efficacy. The results of the conservation analysis by BLASTp, as shown in **Table 1**, indicate that all selected proteins are conserved at 99%–100% with the recorded protein sequences in the NCBI BLASTp database.

**Table 1:** Conservativity of FimH, LptD, and BamA among *E. coli* pathotypes.

Protein	Conservativity percentage (%)
FimH	99.33–100
LptD	99.87–100
BamA	99.88–100

To confirm epitope detection by immune cells, the present study selects only exposed domains characterised by extracellular and outer-membrane features. The results reveal the presence of two exposed domains of FimH: only the domains located between 50–130 a.a. and 160–300 a.a. are selected and further addressed. Both the whole 784 a.a LptD and 809 a.a BamA sequences are identified as outer membrane-exposed domains and are selected for further evaluation.

#### Selection and evaluation of vaccine epitopes

The chimeric vaccine in the present work includes B-cell, CTL, and HTL epitopes. The bioinformatics analysis identified only 14 predicted B-cell epitopes of FimH; of these, 13 failed the evaluation, and 4 were non-antigenic. In comparison, nine epitopes were non-soluble; only one predicted B-cell epitope of FimH, with an antigenicity score of 1.6925 and a conservation percentage of 99.75% (**Table 2**; **Supplementary Figure 1**), was selected and included in the final construct. The prediction analysis identified 76 B-cell epitopes of LptD; 23 were non-antigenic, 23 were poorly soluble, and 17 were allergenic; all were eliminated from the final construct. Only one epitope, with the highest antigenicity score (1.2113) and a conservativity of 98.5% (**Table 2**; **Supplementary Figure 2**), amongst the 13 evaluated B-cell epitopes of LptD, was included in the final construct. On the other hand, 78 B-cell epitopes were identified in the BamA epitope prediction process; only 8 passed the evaluation, and only 1, with the highest antigenicity score (1.5434) and a conservation percentage of 99.82% (**Table 2**; **Supplementary Figure 3**), was included in the final construct. Overall, three B-cell epitopes were selected for the final construct (**Table 2**).

**Table 2:** Final B-cell epitopes of the candidate vaccine.

Protein	Predicted epitopes	Position	Score	Antigenicity	Toxin	Solubility	Allergenicity	Conservation (%)
FimH	PTGGCDASARDVTVTL	9	0.81	1.6925	Non	Good	Non	99.75
LptD	DEHPNDDSSRRWLFYW	300	0.85	1.2113	Non	Good	Non	98.50
BamA	TRWGYGDGLGGKEMPF	632	0.87	1.5434	Non	Good	Non	99.82

- Letters of epitopes refer to one of the 20 amino acids: A, alanine, R, arginine, N, asparagine, D, aspartic acid, C, cystine, Q, glutamine, E, glutamic acid, G, glycine, H, histidine, I, iso-leucine, L, leucine, K, lysine, M, methionine, F, phenylalanine, P, proline, S, serine, T, threonine, W, tryptophan, Y, tyrosine, V, valine.

- Epitope prediction score = 0.5.

- Antigenicity was predicted at 0.4 score.

The prediction analysis also includes the estimation of cytotoxic T cell (CTL) epitopes. The results displayed 122 human and 145 mouse CTL epitopes. A total of 31 human and 35 mouse CTL epitopes of FimH protein were predicted, and amongst them, only 1 human CTL epitope targets human HLA-A*03:01 allele, characterised by a high conservation percentage (99.75%) (**Table 3**; **Supplementary Figure 1**) and antigenicity score (2.0777) with good solubility and evaluated bioinformatically as a non-toxin and non-allergen and, therefore, included in the final MEV construct; the other human and mouse FimH CTL epitopes failed the evaluation process and were excluded from the final construct. The bioinformatic results reveal more CTL epitopes for both LptD and BamA. The results give 36 human and 52 mouse LptD CTL epitopes. From these, four epitopes with a minimum conservation of 93.56% (**Table 3**; **Supplementary Figure 2**) were selected and included in the final construct, targeting human HLA-A*03:01, HLA-A*01:01, and HLA-A*02:01, as well as one mouse CTL epitope designed for the H-2Ld allele with a conservation percentage of 93.11%. On the other hand, the results of CTL epitope prediction give 55 human and 58 mouse epitopes; three BamA epitopes for human HLA alleles passed all the criteria and exhibited conservation percentages that ranged between 99.75% and 99.84% (**Table 3**; **Supplementary Figure 3**) designed for three human HLA alleles (HLA-A*03:01, HLA-A*01:01, and HLA-A*02:01), whilst only one mouse CTL epitope conserved at 99.35% that targets mice H-2-Ld and H-2-Dd was included in the final construct.

**Table 3:** Final cytotoxic T-cell epitopes of the candidate vaccine.

Protein	HLA allele	Start	End	Mer	Epitope	Percentile	Immunogenicity	Antigenicity	Toxin	Solubility	Allergenicity	Conservation (%)
FimH (Human)	HLA-A*03:01	134	142	9	SSAGGVAIK	0.26	0.22944	2.0777	None	Good	Non	99.75
FimH (Mouse)	Failed											
LptD (Human)	HLA-A*03:01	142	150	9	MVGRQGRGK	0.68	0.04566	1.6461	None	Good	Non	100
	HLA-A*01:01	506	514	9	QTLEPRAQY	0.15	0.10041	1.2333	None	Good	Non	98.22
	HLA-A*02:01	507	515	9	TLEPRAQYL	0.47	0.00865	0.9215	None	Good	Non	98.66
	HLA-A*02:01	473	481	9	KLDESVNRV	0.01	0.01049	0.7955	None	Good	Non	93.56
LptD (Mouse)	H-2-Ld	99	107	9	EPVRTVDAL	0.66	0.18372	0.6629	None	Good	Non	93.11
BamA (Human)	HLA-A*01:01	565	573	9	KTDDFTFNY	0.01	0.27508	1.7447	None	Good	Non	99.75
	HLA-A*03:01	609	617	9	KVTLDTATY	0.61	0.11528	0.7757	None	Good	Non	99.84
	HLA-A*01:01					0.51						
	HLA-A*02:01	543	551	9	AMWRYLYSM	0.42	0.01016	1.1855	None	Poor	Non	99.84
BamA (Mouse)	H-2-Ld	117	125	9	SGVRVGESL	0.72	0.12542	0.9114	None	Good	Non	99.35
	H-2-Dd					0.24						

- Letters of epitopes refer to the one of the 20 amino acids: A, alanine, R, arginine, N, asparagine, D, aspartic acid, C, cystine, Q, glutamine, E, glutamic acid, G, glycine, H, histidine, I, iso-leucine, L, leucine, K, lysine, M, methionine, F, phenylalanine, P, proline, S, serine, T, threonine, W, tryptophan, Y, tyrosine, V, valine.

- Epitopes with percentile ≤0.5 are strong binders, those between 0.5 and 2 are weaker binders, and those above 2 are no binders.

- Immunogenicity cutoff value is 0 (>0 considered immunogen whilst <0 are non-immunogens).

- Antigenicity was predicted at 0.4 score.

The results show only 12 helper T cell (HTL) epitopes of FimH; none of these epitopes meets the selection criteria during the immunoinformatic screening and either exhibit no inducer to IL-4 or INF-γ or are non-antigenic, non-soluble, or allergenic; thus, FimH HTL epitopes were completely excluded from the final construct. HTL screening of LptD identified 79 human HTL epitopes; only one 15-mer epitope for HLA-DQA1*01:01/DQB1*02:01 was an inducer of both IL-4 and INF-γ, had a good antigenicity score and conservation (98.75%) (**Table 4**; **Supplementary Figure 2**), was screened as non-toxic and non-allergenic, and was included in the final human HTL epitopes. Also, the screening process identified 18 mouse HTL epitopes from the LptD protein, and 2 met all selected criteria, were conserved at least to 99.23% (**Table 4**; **Supplementary Figure 2**), and were included in the final construct. A total of 60 human and 10 mouse HTL BamA epitopes were predicted, and 1 human HTL epitope for HLA-DQA1*01:01/DQB1*02:01 and 1 mouse HTL epitope for H2-Ied (both conserved at 99.90% as shown in **Supplementary Figure 3**) were included in the final construct (**Table 4**). The results of the present study demonstrate that the candidate vaccine can cover over 77.3% of the global population. As shown in **Table 5**, the HLA-A*02:01 allele is most covered by the selected epitopes.

**Table 4:** Final helper T-cell epitopes of the candidate vaccine.

Protein	HLA allele	S	E	Mer	Epitope	Percentile	Antigenicity	Toxin	Solubility	Allergen	IL-4	IFN	Conservation (%)
FimH (Human)	Failed												
FimH (Mouse)	Failed												
LptD (Human)	HLA-DQA1*01:01/DQB1*02:01	335	349	15	SDPSYFNDFDNKYGS	0.82	0.4004	Non	Good	Non	Inducer	Positive	98.75
LptD (Mouse)	H2-IEd	138	152	15	GDYQMVGRQGRGKAD	0.76	1.2548	Non	Good	Non	Inducer	Negative	99.61
	H2-IEd	252	269	15	MDATITPHYMHRRGN	0.94	1.0752	Non	Good	Non	Inducer	Negative	99.23
BamA (human)	HLA-DQA1*01:01/DQB1*02:01	786	800	15	YAQPFKKYDGDKAEQ	0.97	0.7162	Non	Good	Non	Inducer	Positive	99.90
BamA (Mouse)	H2-IEd	343	357	15	GNRFYVRKIRFEGND	0.79	0.8408	Non	Good	Non	Inducer	Positive	99.90

- Letters of epitopes refer to the one of the 20 amino acids: A, alanine, R, arginine, N, asparagine, D, aspartic acid, C, cystine, Q, glutamine, E, glutamic acid, G, glycine, H, histidine, I, iso-leucine, L, leucine, K, lysine, M, methionine, F, phenylalanine, P, proline, S, serine, T, threonine, W, tryptophan, Y, tyrosine, V, valine.

- Epitopes with percentile ≤2 are strong binders, those between 2 and 10 are weaker binders, and those above 10 are no binders.

- Antigenicity was predicted at 0.4 score.

**Table 5:** Population coverage of the candidate vaccine.

Epitope	Coverage	HLA allele (genotypic frequency (%))					Total HLA hits
	Class I and II	HLA-A*01:01 (9.08)	HLA-A*02:01 (21.95)	HLA-A*03:01 (8.79)	HLA-DQA1*01:01 (9.75)	HLA-DQB1*02:01 (12.27)	
SSAGGVAIK	16.81%	–	–	+	–	–	1
MVGRQGRGK	16.81%	–	–	+	–	–	1
QTLEPRAQY	17.34%	+	–	–	–	–	1
TLEPRAQYL	39.08%	–	+	–	–	–	1
KLDESVNRV	39.08%	–	+	–	–	–	1
KVTLDTATY	17.34%	+	–	–	–	–	1
AMWRYLYSM	39.08%	–	+	–	–	–	1
SDPSYFNDFDNKYGS	18.56%	–	–	–	+	–	1
SDPSYFNDFDNKYGS	23.04%	–	–	–	–	+	1
YAQPFKKYDGDKAEQ	18.56%	–	–	–	+	–	1
YAQPFKKYDGDKAEQ	23.04%	–	–	–	–	+	1
Epitope set	77.30%	2	3	2	2	2	11

- Letters of epitopes refer to the one of the 20 amino acids: A, alanine, R, arginine, N, asparagine, D, aspartic acid, C, cystine, Q, glutamine, E, glutamic acid, G, glycine, H, histidine, I, iso-leucine, L, leucine, K, lysine, M, methionine, F, phenylalanine, P, proline, S, serine, T, threonine, W, tryptophan, Y, tyrosine, V, valine.

### Final construction and characterisation of the candidate vaccine

The primary structure of the chimeric vaccine, as shown in **Supplementary Figure 4**, starts with methionine followed by both β-defensin and PADRE sequences as self-adjuvants at the N-terminal connected by EAAK as an internal linker. In addition, EAAK links the PADRE adjuvant and the first CTL epitope, GGGS linkers bind internal CTL epitopes, the GPGPG links the last CTL epitope with the first HTL epitopes, which is linked by GPGPG, whilst the KK linker links the last HTL epitope with the first B-cell cells, which also link other B-cell epitopes; the construct terminates with poly-histidine (6H) at the C-terminal.

### Secondary and tertiary structures, and validation of the final chimeric protein

**Figure 1:**
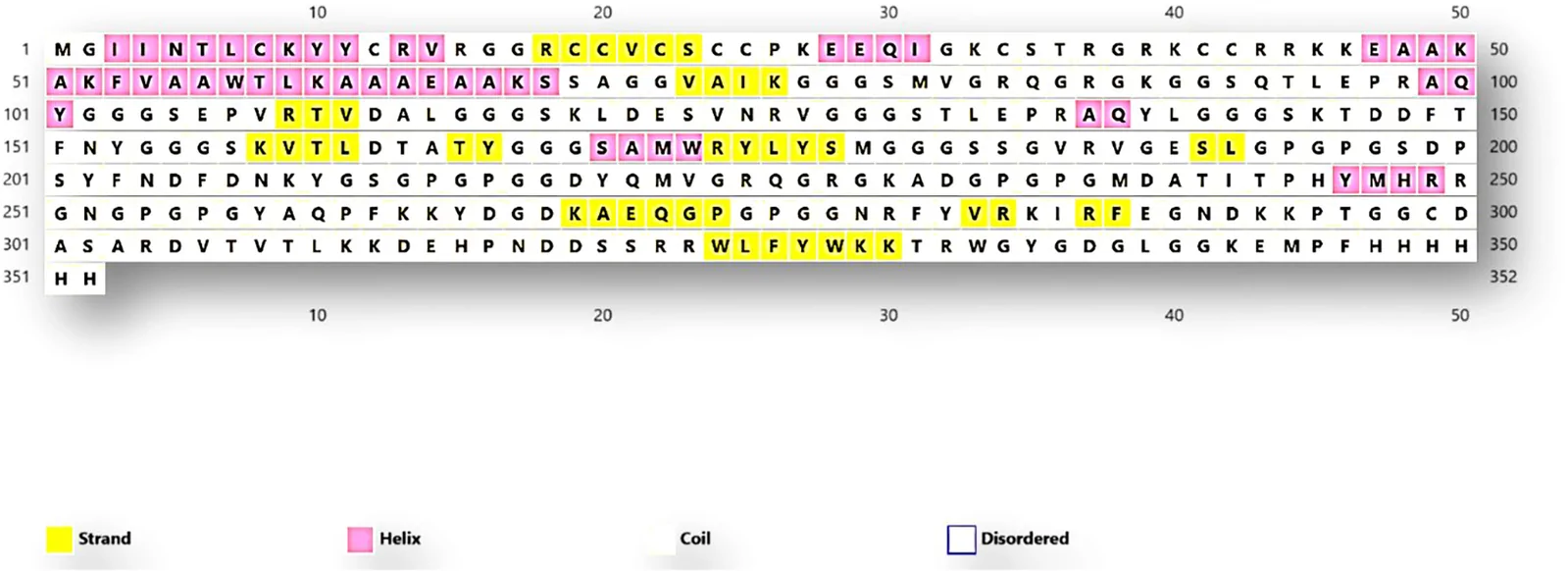
Proposed secondary structure of the chimeric protein using the PESIPRED online server. The structure reveals coils at 82.67%, strands at 12.5%, and helices at 4.83%.

**Figure 2:**
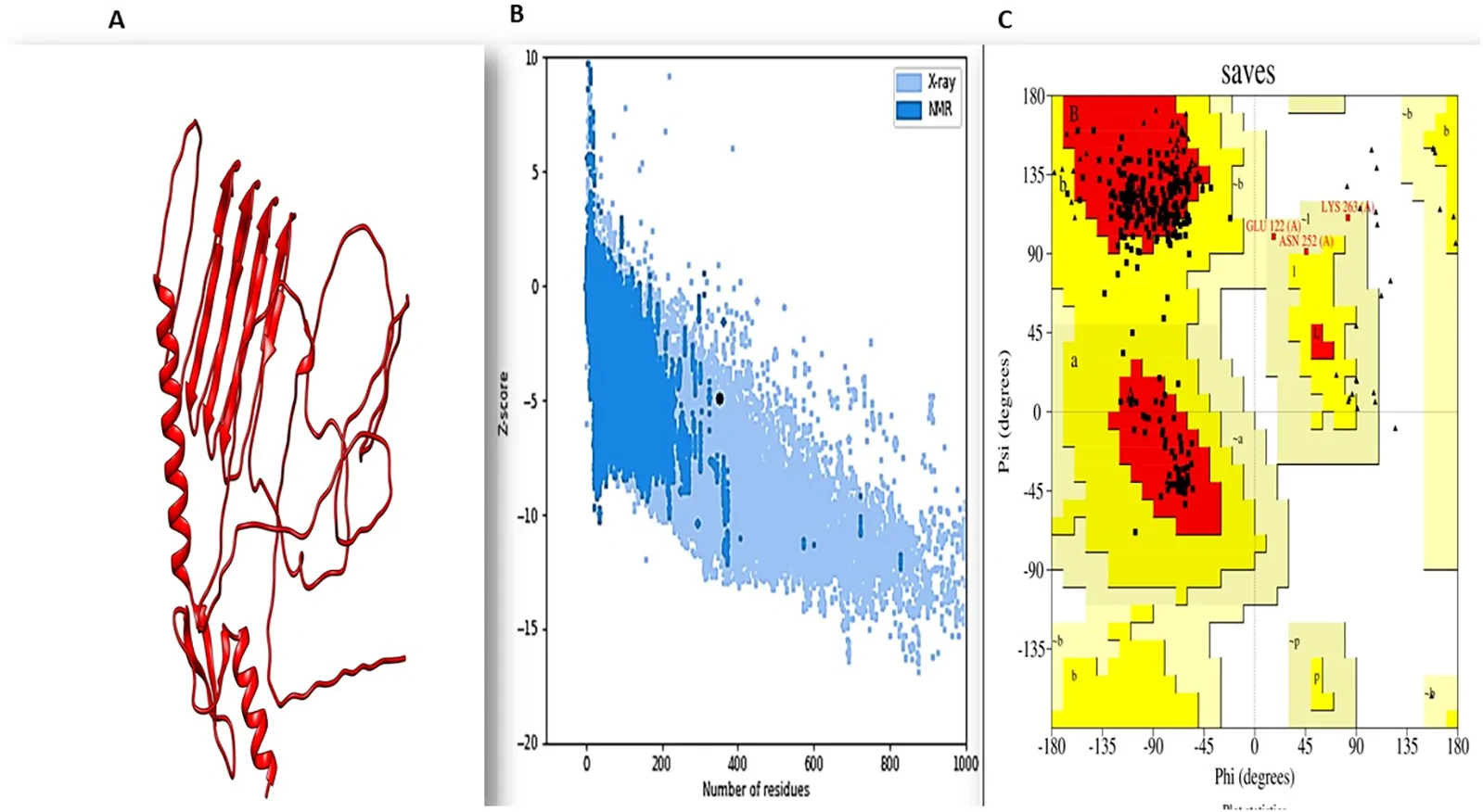
Analysis of the proposed 3D structure of the chimeric protein. **(A)** 3D tertiary structure of the chimeric protein by AlphaFold3, refined by Galaxy Refine. **(B)** *Z*-score of the chimeric protein by ProSA. **(C)** Stereochemical quality by PROCHEK.

The secondary structure, as shown in **Figure 1**, reveals an abundance of coils by 82.67% in the final construct, and the presence of helices and strands in small proportions. The 3D tertiary structure of the candidate vaccine, illustrated in **Figure 2A**, was further refined using Galaxy Refine. The refined 3D structure was further validated using PROCHEK, and the Ramachandran plot (as shown in **Figure 2C**) shows the presence of 86.1% of residues in the favourable region and 14.9% in allowed regions and no residues present (0%) in the disallowed region. Finally, the *Z*-score result from ProsA-web, as shown in **Figure 2B**, was −4.94. Overall validation indicates that the 3D structure is reliable and suitable for docking analysis.

Based on the results of Expasy ProtParam, the physicochemical properties (**Table 6**) show that the construct has a 37,394.77 molecular weight with a theoretical pI of 9.53, and the extinction coefficient of the chimeric protein was 53,455 M^−1^ cm^−1^, with an instability index and hydropathicity of 36.44 and −0.836, respectively, indicating that the candidate MEV is stable and hydrophilic. The chimeric vaccine reveals a high antigenicity score (1.25), good solubility, and non-allergenicity, as listed in **Table 7**. Finally, the similarity between the candidate MEV and the protein sequence of *Lactobacillus johnsonii*, a model for human probiotics, was evaluated, and the results indicate no similarity between the two, revealing that the candidate chimeric protein is safe and that the elicited immune responses do not affect human probiotics.

**Table 6:** Physicochemical properties of the chimeric vaccine by the Expasy ProtParam tool.

Property	Result
Number of amino acids	352
Theoretical pI	9.53
Molecular weight (Dalton)	37,394.77
Extinction coefficients (M^−1^ cm ^−1^)	53,455
Estimated half-life (h)	More than 10 in *E. coli* (*in vivo*)
Instability index	36.44
Hydropathicity (GRAVY)	−0.836

**Table 7:** Antigenicity, solubility, and allergenicity evaluation of the candidate vaccine.

Property	Result
Antigenicity score	1.2510
Water solubility	Good water solubility
Allergenicity	Non-allergen

### Molecular docking of the chimeric protein with TLR4

**Figure 3:**
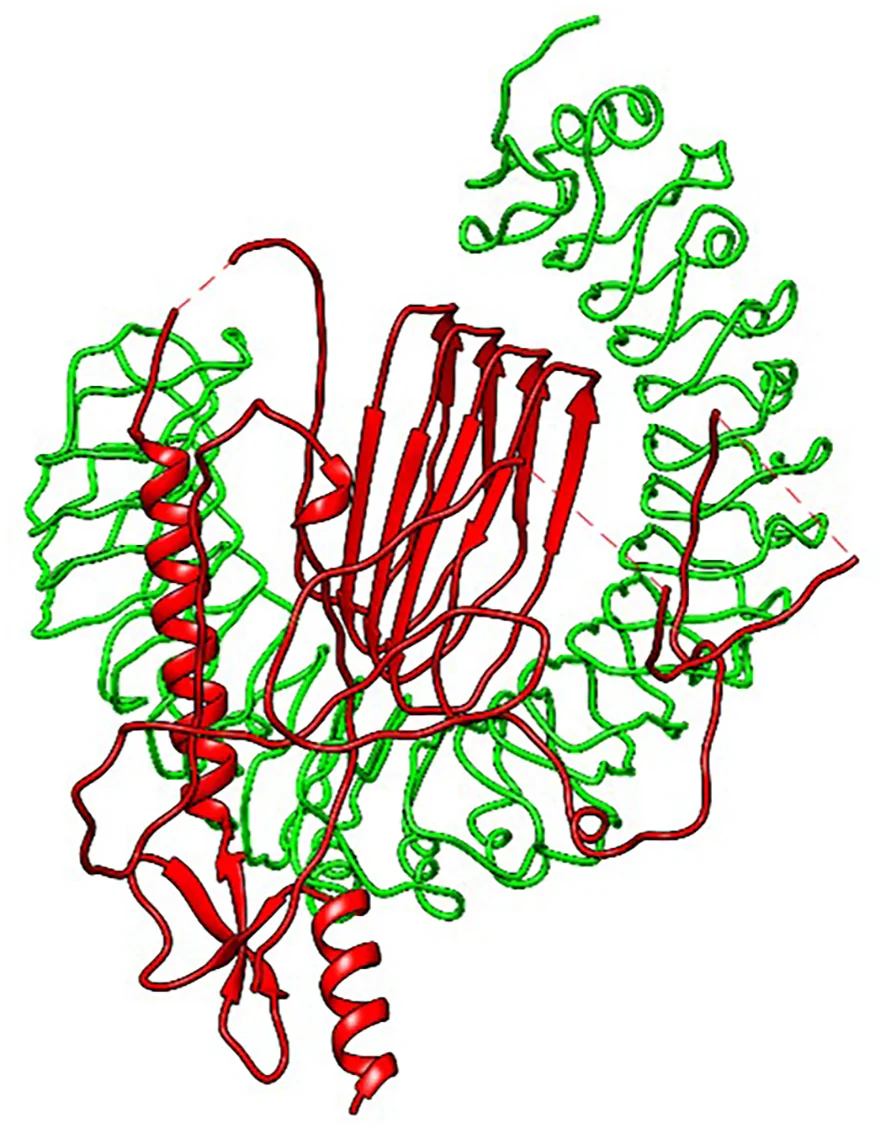
Docked complex between the candidate vaccine and human TLR-4. The green chain is TLR-4; the red chain is the candidate vaccine.

Many docked models were generated from HDOCK. The best model (model 0) was further evaluated. The docking between MEV and TLR-4 is illustrated in **Figure 3**. **Table 8** shows 17 hydrogen bonds between the MEV and the TLR-4 receptor.

**Table 8:** Intermolecular hydrogen bonding between the candidate chimeric vaccine and TLR-4.

No.	TLR-4	Chimeric vaccine	Number of hydrogen bonds
	Amino acid (position)	Amino acid (position)	
1.	Arginine (246)	Glycine (142)	2
2.	Threonine (359)	Lysine (119)	2
3.	Serine (360)	Lysine (119)	1
4.	Asparagine (361)	Lysine (145)	1
5.	Asparagine (409)	Arginine (174)	1
6.	Asparagine (433)	Aspartic acid (219)	2
7.	Arginine (460)	Glutamine (221)	1
8.	Glutamine (505)	Asparagine (152)	1
9.	Glutamine (505)	Serine (178)	1
10.	Glutamine (578)	Valine (127)	1
11.	Glutamine (578)	Glycine (129)	1
12.	Glutamine (578)	Glycine (154)	1
13.	Arginine (606)	Glutamine (100)	1
14.	Glutamic acid (608)	Glycine (103)	1

### Codon optimisation, *in silico* cloning, and tandem repeats screening

**Figure 4:**
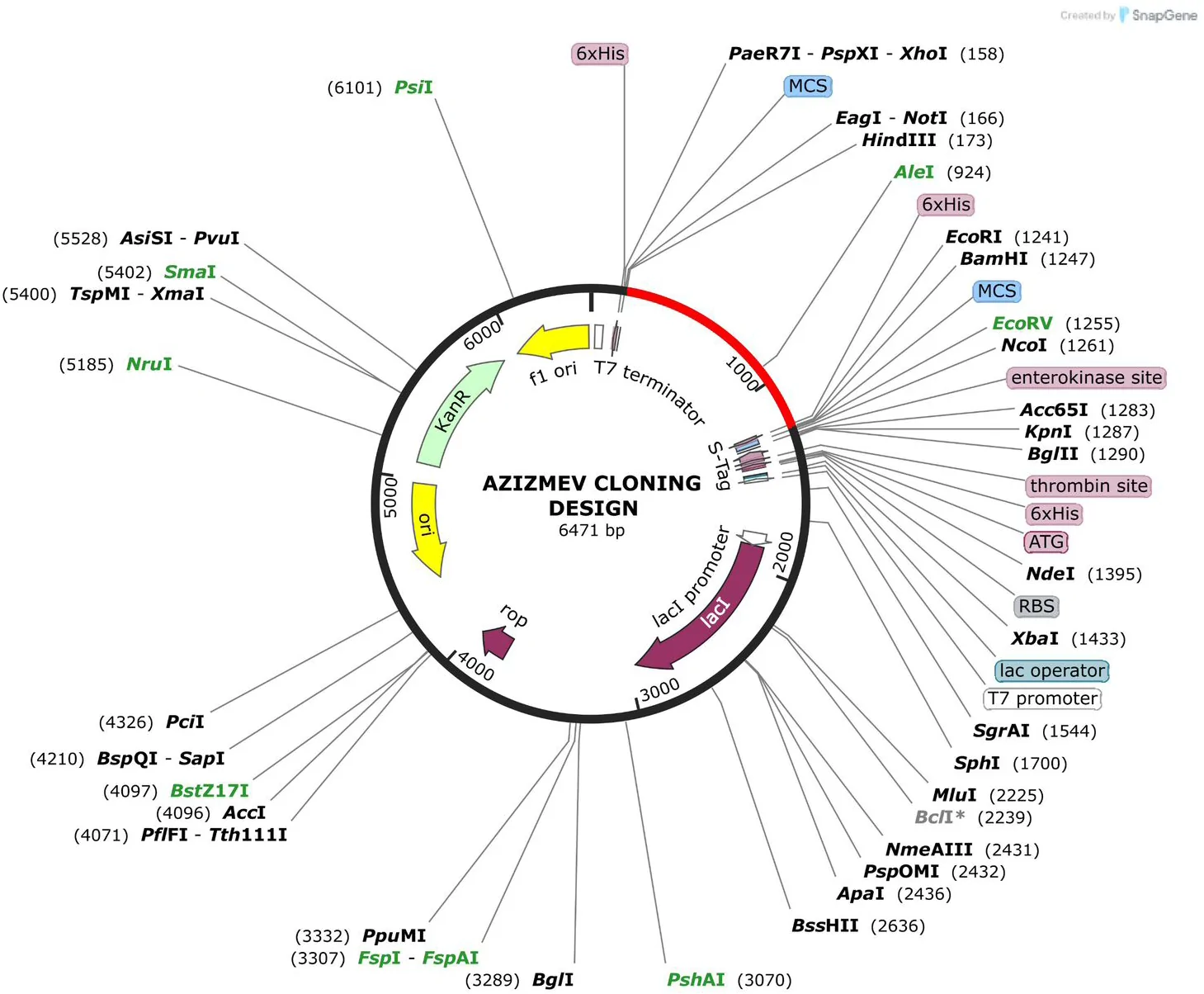
*In silico* cloning and expression vector map of the candidate chimeric vaccine by SnapGene software. The target fragment (red highlighted) was inserted into the multiple cloning site, with a 5′ flanking restriction site at (173) for EcoR1 and a 3′ flanking site at (1241) for HindIII.

Gene expression is a crucial step for effective vaccine development. Before protein expression, this study identified codons for the candidate vaccine designed for expression in the *E. coli* BL21 system. The Jcat online tool was used to determine a CAI value of 1; the resulting codon is shown in **Supplementary Data Sheet 1**. The codons start with ATG, specific for methionine, to confirm gene expression, and end with six CAC codons, specific for histidine, to confirm ease of extraction and purification of the target protein by affinity chromatography. The optimised codons exhibit a GC content of 53.598%, suggesting effective expression. Before the cloning step, the optimised codon was further evaluated to exclude tandem repeats. Finally, the mRNA sequence was cloned into the pET30a expression vector, as illustrated in **Figure 4**, yielding a final molecular weight of 6,471 bp.

### Experimental evaluation of the proposed chimeric vaccine

#### Expression and purification of the candidate MEV

**Figure 5:**
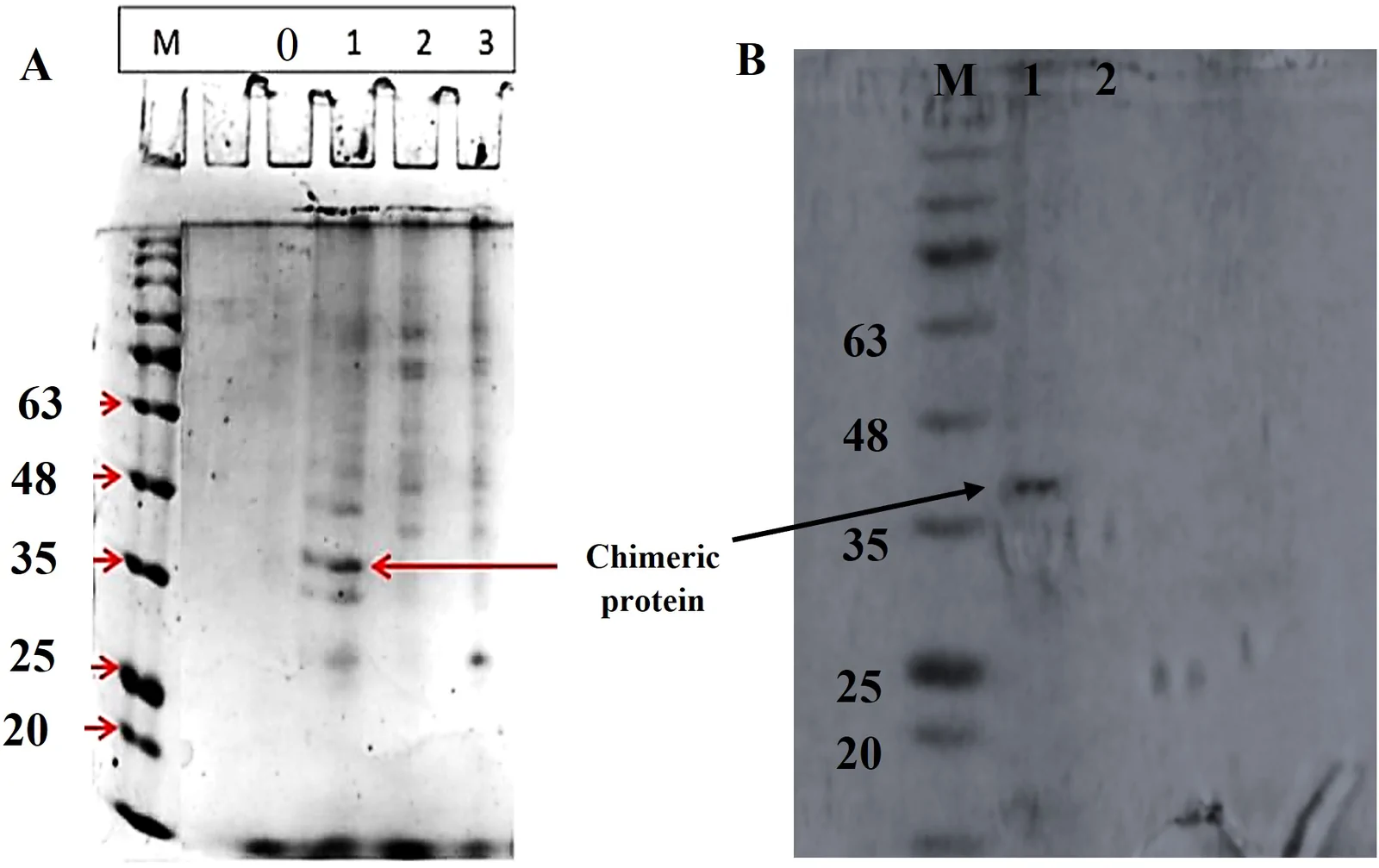
Purification and detection of the chimeric protein. The chimeric protein from the construct was purified using nickel resins. **(A)** SDS-PAGE of induced expression, protein marker (M), denaturing condition (lane 0), supernatant of the induced expression with 1 mM IPTG (lane 1), crude extract of non-induced induction (lane 2), and negative control (lane 3). **(B)** SDS-PAGE of purification, protein marker (M), elution treated with 500 mM imidazole (lane 1), and wash part (lane 2).

The synthetic gene was cloned into the pET-30a expression vector (Macrogen/Korea). Transformation was performed using a heat-shock protocol using competent *E. coli* (BL21), then the cells were cultured on LB agar with kanamycin. Many colonies were obtained from the transformation. The SDS-PAGE analysis shows that the candidate chimeric protein is present in the supernatant (**Figure 5A**), indicating that it is soluble and does not form inclusion bodies. Further evaluation shows that successful purification of the chimeric protein via affinity chromatography is achieved when treated with 500 mM imidazole (**Figure 5B**).

### Stimulation activity of the chimeric vaccine on humoral immune responses

**Figure 6:**
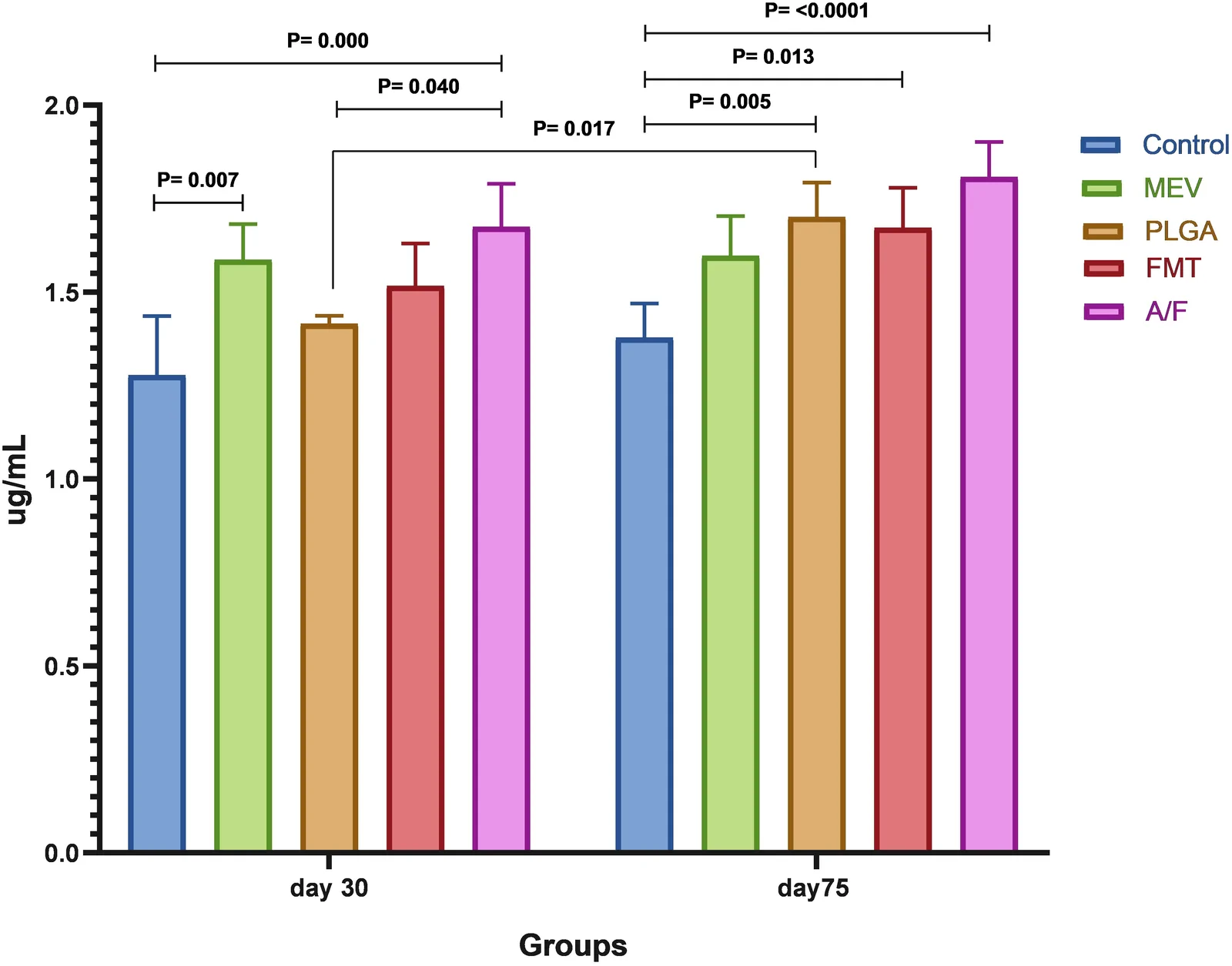
IgG1 responses stimulated by different vaccine formulations. MEV represents mice immunised with the chimeric vaccine only; PLGA represents mice immunised with purified PLGA-encapsulated chimeric vaccine; FMT represents immunised mice with the chimeric vaccine after FMT; A/F represents immunised mice with the chimeric vaccine after FMT combined with gut microbiota modulation. The graph explains IgG1 titres quantified by ELISA at both 30 and 75 days after the first immunisation dose of the chimeric vaccine. Each tested group contains a sample size *n* = 5. The differences were evaluated between all tested groups, and *p*-values below 0.05 were considered significant. Bars represent mean ± SD of mice per group.

The chimeric vaccine of the present study displays a variation in its immunogenicity; as shown in **Figure 6** and **Supplementary Table 1**, the MEV alone causes a significant (*p* = 0.007) increase in the IgG1 levels after 30 days from the first dose, accounting for (1.59 ± 0.09) µg/mL compared to (1.28 ± 0.16) µg/mL in the control mice treated with PBS. At the same immunisation time, the immunised mice group with both PLGA-encapsulated MEV and purified MEV after FMT without gut microbiota modulation do not exhibit significant differences (*p* > 0.05) in IgG1 levels. In contrast, combining FMT with gut microbiota modulation using cocktail antibiotics was associated with the highest significant (*p* = 0.000) increase in IgG1 (1.67 ± 0.12 µg/mL) compared with the PLGA-encapsulated MEV-treated and control groups. Likewise, these immunised mice exhibit a greater (*p* ≤ 0.0001) increase in IgG1 levels at 75 days (1.81 ± 0.09 µg/mL) compared with the control group. Only mice immunised with PLGA-encapsulated MEV showed higher (*p* = 0.017) IgG1 levels at 72 days than at 30 days.

**Figure 7:**
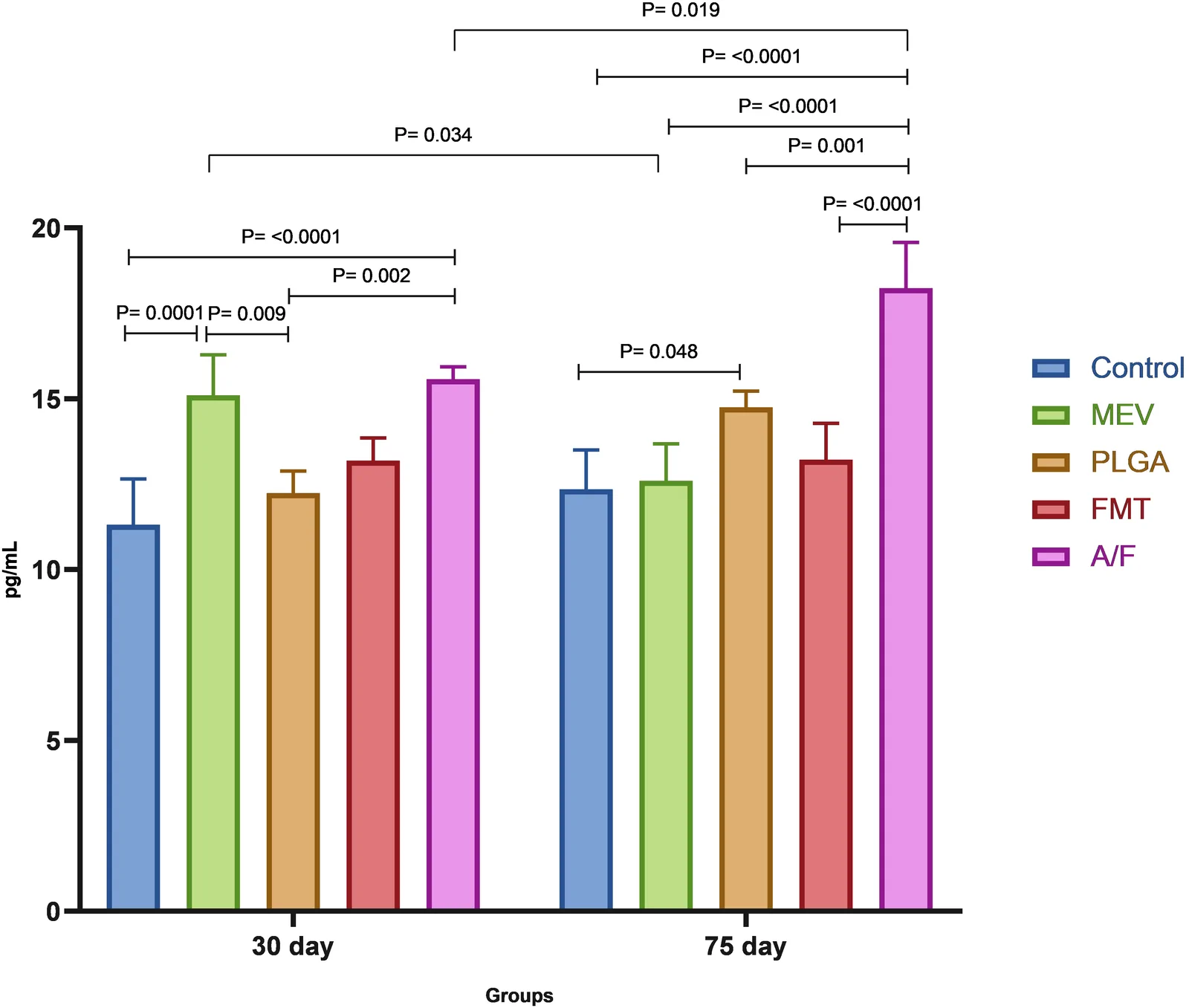
IL-4 responses stimulated by different vaccine formulations. MEV represents mice immunised with the chimeric vaccine only; PLGA represents mice immunised with purified PLGA-encapsulated chimeric vaccine; FMT represents immunised mice with the chimeric vaccine after FMT; A/F represents immunised mice with the chimeric vaccine after FMT combined with gut microbiota modulation. IL-4 titres measured by ELISA at 30 and 75 days following the initial dose of the chimeric vaccine are explained in the graph. The sample size is *n* = 5 in each tested group. All differences between groups were assessed, and *p*-values < 0.05 were deemed significant. Bars show the mice's mean ± SD for each group.

As shown in **Figure 7** and **Supplementary Table 1**, the highest significant (*p* < 0.05) elevation in IL-4 levels was seen in the serum of immunised mice with chimeric protein after FMT with gut microbiota modulation, accounting for (18.24 ± 1.34) pg/mL and (15.58 ± 0.36) pg/mL at both tested times, respectively. In comparison, other formulations of the chimeric protein, including MEV alone and PLGA-encapsulated MEV, showed a short-term ability to induce IL-4, with a limited effect lasting only 30 days. At the same time, immunisation with MEV after FMT without gut microbiota modulation did not show a significant effect (*p* > 0.05).

**Figure 8:**
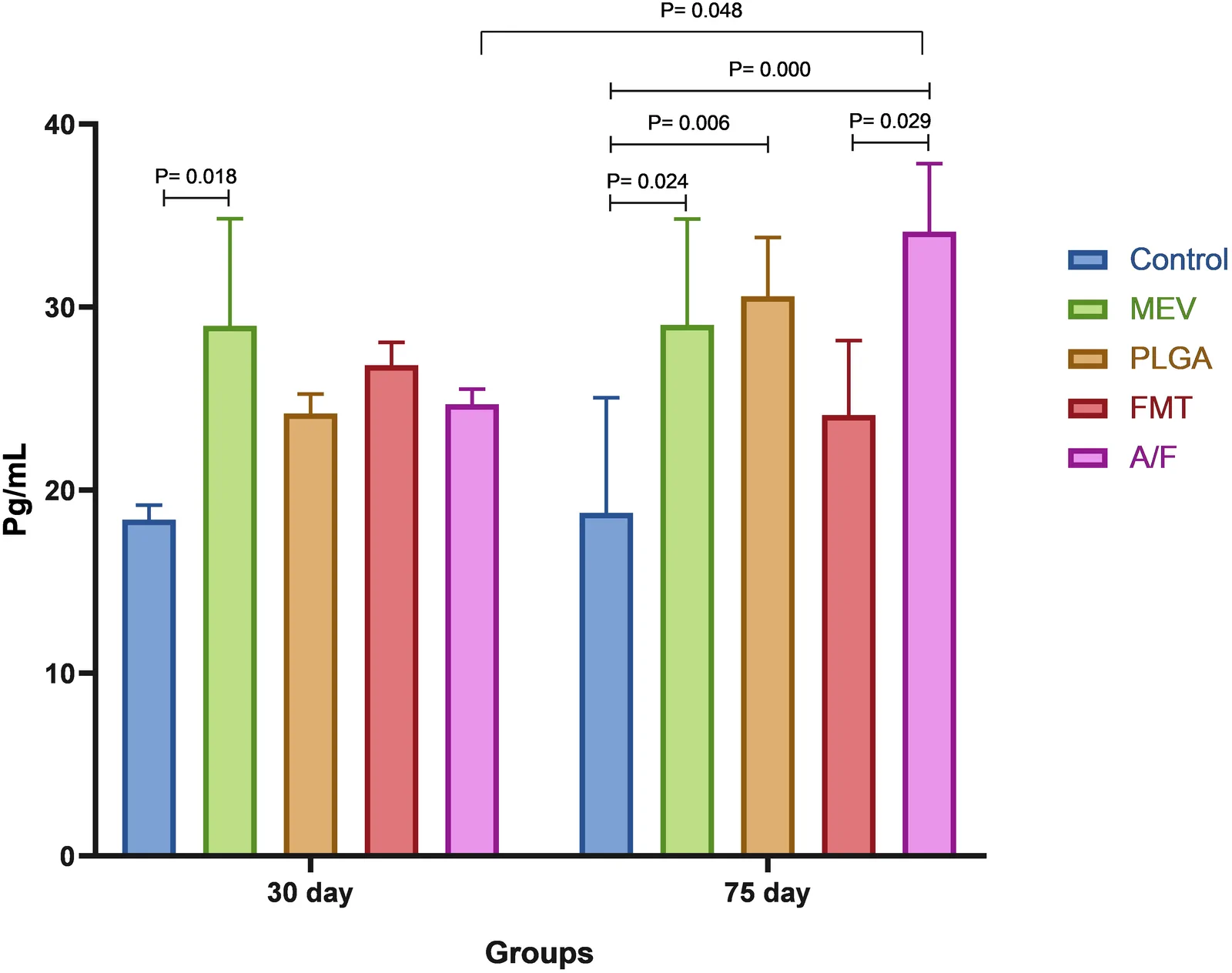
INF-γ responses stimulated by different vaccine formulations. MEV represents mice immunised with the chimeric vaccine only; PLGA represents mice immunised with purified PLGA-encapsulated chimeric vaccine; FMT represents immunised mice with the chimeric vaccine after FMT; A/F represents immunised mice with the chimeric vaccine after FMT combined with gut microbiota modulation. Bars represent mean ± SD from five mice per group; all *p*-values below 0.05 represent significant differences.

Immunogenicity of the chimeric vaccine in the induction of INF-γ demonstrated a delayed onset (**Figure 8**; **Supplementary Table 1**), as only immunisation with purified chimeric protein alone causes a significant (*p* = 0.018) increase in INF-γ level, amounting to (28.98 ± 5.84) pg/mL compared to (18.39 ± 0.78) pg/mL in the control group. In contrast, a robust, significant increase became evident after 75 days. Most formulations at this time provoke a significant increase in INF-γ levels, including immunisation with the MEV alone (29.02 ± 5.79 pg/mL, *p* = 0.024) and PLGA-encapsulated MEV (30.59 ± 3.21 pg/mL, *p* = 0.006). Remarkably, formulating the chimeric vaccine with FMT combined with gut microbiota modulation demonstrated a superior capacity (*p* = 0.000) to induce INF-γ at 75 days following the first immunisation dose, accounting for (34.13 ± 3.71) pg/mL compared with the control.

**Figure 9:**
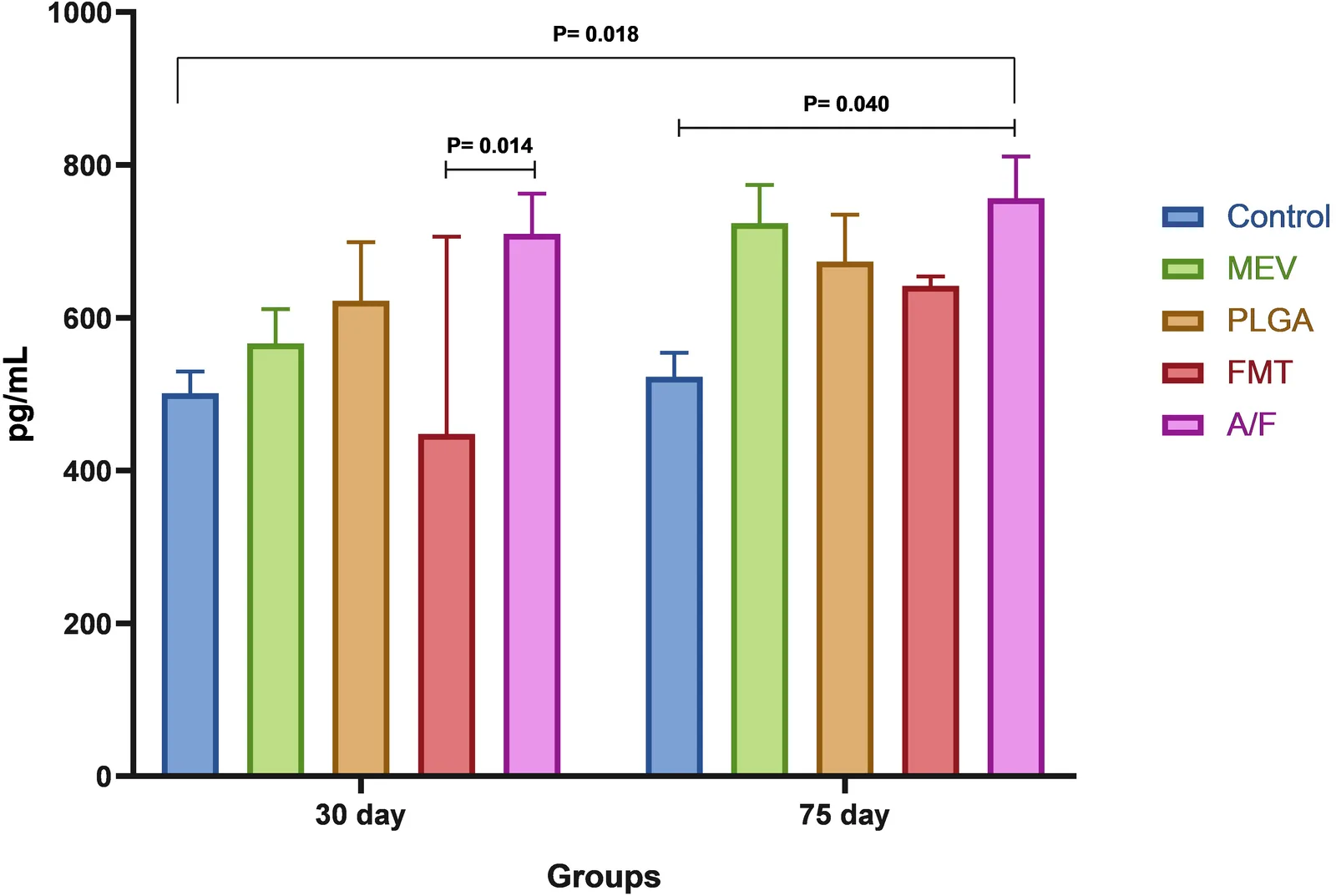
NLRP3 responses stimulated by different vaccine formulations. Mice immunised with the chimeric vaccine alone are represented by MEV; PLGA represents mice immunised with purified PLGA-encapsulated chimeric vaccine; mice immunised with the chimeric vaccine following FMT are represented by FMT; and mice immunised with the chimeric vaccine following FMT in conjunction with gut microbiota modulation are represented by A/F. All *p*-values below 0.05 indicate significant differences; bars show the mean ± SD from five mice per group.

Only immunisation after FMT, combined with modulation of the gut microbiota, significantly enhances (*p* = 0.014 and *p* = 0.040) serum NLRP3 levels at both tested times, as illustrated in **Figure 9** and **Supplementary Table 1**. Serum NLRP2 was elevated to (709.92 ± 52.41) pg/mL at 30 days and (756.52 ± 54.73) pg/mL at 75 days compared to the control groups. Other chimeric vaccine formulations do not elicit any significant activation (*p* > 0.05) compared to the control group.

### Protection efficacy of the candidate vaccine against B2 UPEC

**Figure 10:**
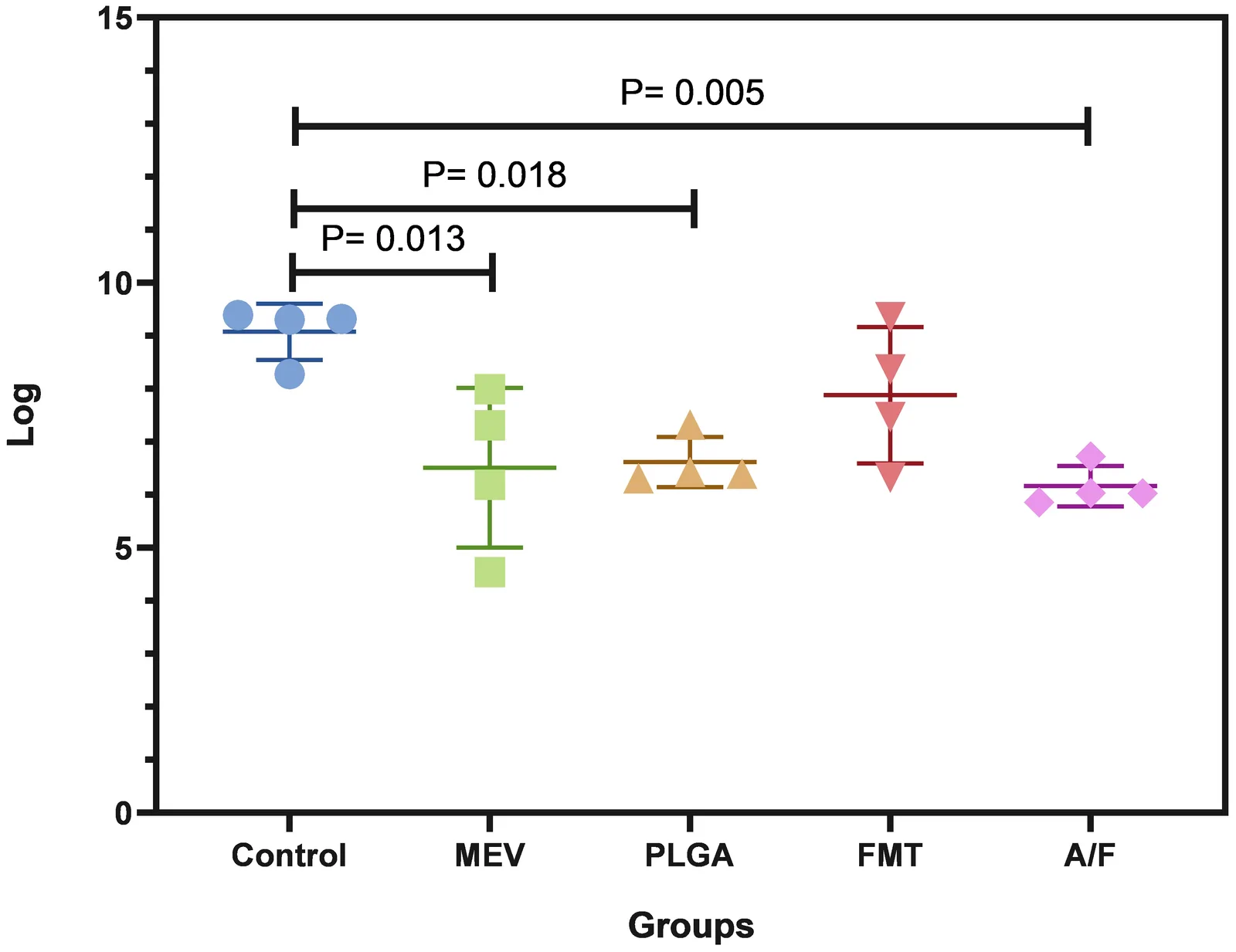
Log reduction of bacterial load in mice bladders stimulated by different vaccine formulations. Mice immunised with the chimeric vaccine alone are represented by MEV; PLGA represents mice immunised with purified PLGA-encapsulated chimeric vaccine; FMT represents mice immunised with the chimeric vaccine following FMT; and mice immunised with the chimeric vaccine following FMT in conjunction with gut microbiota modulation are represented by A/F. Each tested group includes a sample size of *n* = 4. Data are represented as mean ± SD significant differences represented by *p*-values below 0.05.

The candidate vaccine in the present study, targeting UPEC as a model for the pathogenesis of *E. coli* pathotypes, showed high efficacy in immunised mice with all MEV formulations, except in those immunised with MEV without gut microbiota modulation. Amongst the tested groups, immunised mice with MEV after FMT combined with gut microbiota modulation exhibited the greatest reduction (*p* = 0.005) in UPEC load, with an 813-fold decrease compared to PBS-treated mice (as shown in **Figure 10**; **Supplementary Table 2**), followed sequentially by the MEV-alone-immunised mice by a 360-fold bacterial load reduction (*p* = 0.013), then PLGA-encapsulated MEV-immunised mice with a 282-fold reduction (*p* = 0.018).

### Evaluation of the toxicity of the candidate vaccine

**Figure 11:**
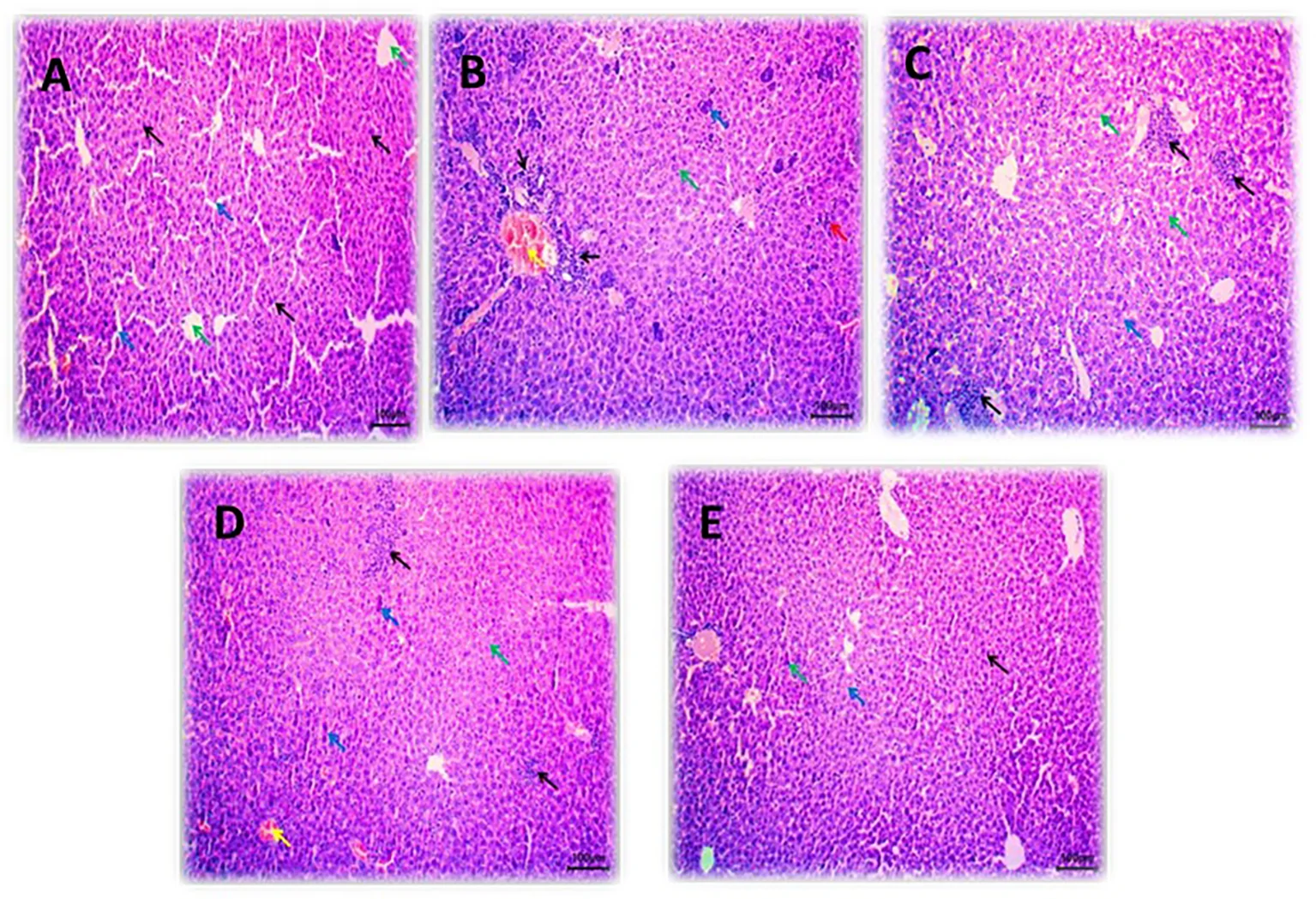
Liver histological effects of the candidate chimeric vaccine. Infiltration of the inflammatory cells represented by black arrows, coagulative necrosis represented by green arrows, degeneration of the hepatocytes represented by blue arrows, and portal vein congestion represented by yellow arrows. Non-immunised mice (control) **(A)**; Mice immunised with the chimeric vaccine alone **(B)**; mice immunised with purified PLGA-encapsulated chimeric vaccine **(C)**; mice immunised with the chimeric vaccine following FMT **(D)**; and mice immunised with the chimeric vaccine following FMT in conjunction with gut microbiota modulation **(E)**.

**Figure 12:**
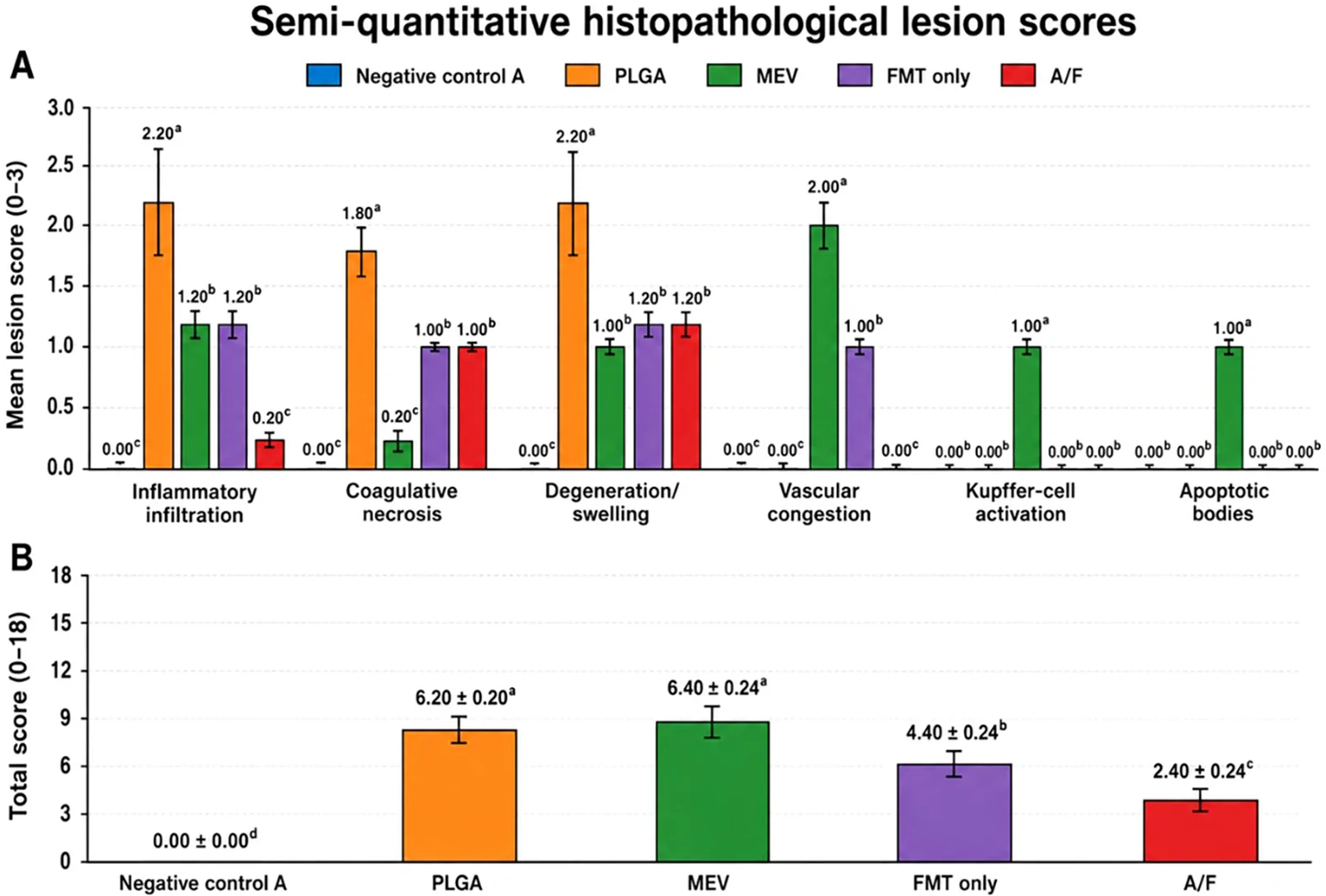
Semi-quantitative histopathological lesion scores in the experimental groups. Bars represent mean ± SEM values. Different superscript letters represent illustrative statistical groupings. A two-sided *p*-value < 0.05 was considered statistically significant. **(A)** Semi-quantitative scoring by an observer blinded to the experimental groups. Inflammatory-cell infiltration, coagulative necrosis, hepatocellular degeneration/swelling, vascular congestion, Kupffer-cell activation, and apoptotic bodies were graded on a 0–3 scale, where 0 indicated absence, 1 denoted mild/focal, 2 indicated moderate/multifocal, and 3 denoted marked/diffuse involvement. **(B)** Provisional semi-quantitative histopathological lesion scores; five non-overlapping microscopic fields were evaluated for each animal at a predefined magnification by an observer blinded to the experimental-group allocation. The median score of the examined fields was used as the animal-level score for each lesion. A composite histopathological score, ranging from 0 to 18, was calculated for each animal by summing the six individual lesion scores.

The PLGA group exhibited evident lesions (**Figure 11**), mainly characterised by inflammatory-cell infiltration, coagulative necrosis, and hepatocellular degeneration or swelling. In contrast, vascular congestion, Kupffer-cell activation, and apoptotic bodies were not observed. Accordingly, the total lesion score reached approximately 6.2, ranking amongst the highest in the experimental groups (**Figure 12**). The MEV group recorded the highest overall lesion score, approximately 6.4 (**Figure 12**); however, its pattern of hepatic injury differed from that observed in the PLGA group. It was characterised by vascular congestion, Kupffer-cell activation, and the presence of apoptotic bodies, along with milder infiltration of inflammatory cells and hepatocellular degeneration. These findings suggest that MEV induced a more diverse spectrum of lesions, particularly vascular and cellular alterations. The FMT-only group exhibited moderately severe histopathological changes, including mild-to-moderate inflammatory infiltration, necrosis, hepatocellular degeneration, and vascular congestion (**Figure 11**). The total lesion score in this group was 4.4 (**Figure 12**). The immunisation after FMT with modulation of gut microbiota showed the mildest lesions amongst the treated groups. The observed alterations were mainly limited to coagulative necrosis and mild hepatocellular degeneration, with no evidence of vascular congestion, Kupffer-cell activation, or apoptotic bodies (**Figure 11**). Consequently, the total lesion score was 2.4 (**Figure 12**), suggesting that FMT combined with modulation of the gut microbiota significantly reduces vaccine side effects.

### Role of FMT-based adjuvant for enhancing vaccine efficacy

Overall, the inclusion of FMT as an adjuvant enhanced the performance of candidate vaccine in terms of multiple evaluation parameters. FMT with gut microbiota modulation induced better humoral and cellular immune responses than the other vaccine formulations, as proved by higher IgG1, IL-4, IFN-γ, and NLRP3 levels. This increased immunogenicity resulted in improved protection against UPEC challenge, as shown by the largest decrease in bacterial load in the bladder. Also, histopathological assessment indicated an acceptable safety profile of the formulation, which further supports the favourable role of FMT in improving the overall efficacy of the candidate vaccine.

## Discussion

Here, in the present study, three proteins commonly associated with the pathogenicity of *E. coli* pathotypes are targeted in the candidate chimeric vaccine. The results show high conservation of all selected proteins and epitopes across *E. coli* pathotypes, suggesting that the candidate vaccine of the present study could provide good protection against *E. coli* pathotypes.

Lipopolysaccharide (LPS) is a major component of the cell wall of Gram-negative bacteria and plays several crucial roles in pathogenesis; it enables bacteria to withstand harsh environments and supports bacterial colonisation. Additionally, recent evidence indicates that LPS plays a role in antibiotic resistance by blocking its entry into cells (52, 53). LPS is synthesised in the cytoplasm and transported to the outer leaflet via several types of outer membrane proteins (OMPs). Thus, inhibition of LPS translocation via targeting one of the OMPs is possible. Amongst these OMPs, LptD is highly immunogenic (54) and mediates the final step in the translocation of LPS into the outer membrane (52). On the other hand, the BamA protein is well documented as a part of the Bam complex in *E. coli* and plays an important role in the assembly of β-barrel proteins into the outer membrane (55). It exhibits strong immunogenicity, making it a promising protective antigen against *Acinetobacter baumannii* infection (56). Furthermore, the recent finding of Soltan et al. (24) indicates that both LptD and BamA are highly conserved amongst Gram-negative bacteria, suggesting that both could be an ideal choice for future promising vaccines; thus, the present work aimed to construct a candidate chimeric vaccine against infections caused by *E. coli* pathotypes using UPEC as a model by combining both Gram-negative conserved LptD and BamA with FimH, which play an important role in the colonisation of UPEC, as well as other Gram-negative bacteria (57).

Involving extracellular domains of selected proteins as components of chimeric vaccines is crucial for inducing efficient immune responses (58); thus, the present study focuses on using exposed extracellular domains as the sole basis for epitope prediction.

Both ABCpred and the IEDB bioinformatic tools were used to predict B-cell, CTL, and HTL epitopes of the selected proteins. ABCpred at a threshold score of 0.4 was used for screening linear B-cell epitopes; the main advantage of this web bioinformatics tool is the use of a neural network algorithm to evaluate the accuracy of B-cell epitopes in terms of selectivity, specificity, and positive predicting (59), and the threshold score of 0.4 is well addressed in several bioinformatic studies (60). On the other hand, IEDB was used to predict both CTL and HTL epitopes. This bioinformatic tool uses artificial neural networks (ANNs) based on experimental binding affinity to human and mice HLA alleles (61), and is an easily accessible online tool and well characterised by well-established validation (62), and because of the aims of the present work that seeks the construction of a chimeric vaccine containing both CTL and HTL epitopes, the IEDB tool is well compatible with this purpose and is increasingly used by previous works (63, 64).

The present work focuses on constructing MEV for locally predominant *E. coli* pathotypes and uses the most locally prevalent UPEC phylogroup, so as to make the designation more compatible with our purpose. Our MEV is designed to contain CTL and HTL epitopes with binding affinity for the most frequent Iraqi HLA alleles. Most HLA alleles in the Iraqi population are used solely as a base criterion for selecting both CTL and HTL epitopes. The candidate vaccine of the present study bioinformatically has a good binding affinity with the three most frequent MHC class I alleles worldwide, namely, HLA-A*03:01, HLA-A*01:01, and HLA-A*02:01, and HLA-DQA1*01:01/DQB1*02:01 of the MHC class II allele, and based on previous local studies (43–46), the most frequent HLA alleles amongst the Iraqi population were HLA-A*02:01 followed by both HLA-A*01:01 and HLA-A*03:01, whereas HLA-DQA1*01:01 is the most frequent HLA allele of MHC class II, and this was further confirmed by the Allele Frequency Net Database (https://www.allelefrequencies.net/hla.asp). Thus, the epitopes included in our chimeric vaccine were suggested to evoke good immune responses amongst people from different geographical areas, and especially amongst Iraqi population.

One of the major problems of *E. coli* is its genetic flexibility and the presence of several pathotypes, which cause extraintestinal and intestinal diseases. Despite several types of vaccines against *E. coli*, there is still no approved vaccine, and this requires conducting more studies to build other safe and promising vaccines able to evoke broad immune responses against all pathotypes; hence, we aimed to use several conserved epitopes across *E. coli* pathotypes capable to evoke both humoral and cellular immune responses against the local UPEC isolate as a model for *E. coli* infections.

The chimeric vaccine of the present work was clearly able to induce antibody responses, as all MEV formulations cause a significant increase in antibody concentration at different tested times; the reason behind this increase may be the immunogenic nature of the three included proteins. It is clearly noted that the purified LptD protein of different Gram-negative bacteria causes an increase in antibody concentration and is considered as a promising target vaccine (65, 66); in addition, BamA of *E. coli* (67) and *A. baumannii* (68) and FimH (69–71) show the same immunogenicity effect.

Overall, the designed vaccine elicits promising humoral immune responses, as most MEV formulations induce strong Th1 and Th2 responses through increased concentrations of both IL-4 and INF-γ, and compared to previous studies, our results are consistent with previous findings (17, 18, 20, 72). Increased Th1 and Th2 responses confirm the recruitment of antigen-presenting cells, CD4 cells, and CD8 cells, indicating that our chimeric protein is immunogenic.

In the present study, the effectiveness of the chimeric vaccine was evaluated using intraperitoneal immunisation. This immunisation route is clearly established as a reliable method in the murine model and effectively elicits strong IgG and IgA responses, providing essential protection against UPEC (73). However, because UTIs are characterised as mucosal infections, we acknowledge that mucosal immunisation could be more relevant. It is well established that mucosal vaccination induces the local production of secretory IgA (sIgA) that directly interferes with the adherence of UPEC to the urothelium (17, 74). Thus, an important avenue for our future investigations is a comparison of mucosal and systemic prime-boost strategies.

We investigated FMT as a new-generation adjuvant for bacterial vaccines. FMT was used in two ways: first, without modulating the gut microbiota; and second, after gut microbiota washout with a cocktail of antibiotics. Vaccination after FMT, without gut microbiota modulation, does not generally affect humoral immune responses. In contrast, after gut microbiota modulation, the outcomes of humoral immune responses were beneficial and significant compared to the non-gut microbiota modulation case or with MEV alone, and PLGA encapsulation highlights its adjuvanticity property in the vaccination process. Here, we hypothesised that this effect may be linked to NLRP3 and, depending on the results, NLRP3 levels. Vaccination after FMT with gut microbiota modulation significantly increases NLRP3 levels, especially compared with levels in a group receiving FMT without modulation.

The exact underlying mechanism of FMT’s adjuvant effect in the present work is not well addressed. However, earlier evidence investigated the role of SCFAs in UTIs. SCFA-producing bacteria reduce bacterial load in the bladder via an unknown mechanism (75). Likewise, other evidence indicates a positive correlation between SCFA and NLRP3 (40). Thus, we thought that the FMT after gut modulation in the present work improves the abundance of SCFA-producing probiotic bacteria, which, in turn, enhances activation of the NLRP3 inflammasome.

Several previous investigations indicate that UTI caused by UPEC is amongst the most prevalent UTI-causing bacteria and poses a major health risk worldwide (76). Antibiotic therapy is considered the first-line treatment. However, this bacterium shows a high rate of antibiotic resistance, highlighting the need to develop alternative therapies (76, 77), making vaccination a viable option for UTIs.

To date, several candidate vaccines for UPEC have been developed, including Urovac, Uro-Vaxom, Urvakol, and UROtism. However, several limitations of these vaccines, such as side effects (e.g., allergic reactions), daily doses required, and short-term protection, restrict their use (78). Hence, the subunit vaccine is an effective and safe approach for treating several bacterial infections (79–81).

The most predominant and highly antibiotic-resistant local *E. coli* phylogroup (B2) was used as a model of infection. The results show a good outcome, as all MEV formulations, except after FMT without gut modulation, provide effective bacterial protection, with at least a 261-fold reduction, and this is in agreement with previous studies that used FimH as one subunit of MEV (17–19, 72), as well as with more recent works dependent on FimH as a major vaccine component (71, 82).

To design a candidate vaccine targeting all *E. coli* pathotypes, we rely on three subunit proteins, both LptD and BamA, bioinformatically identified as the most conserved amongst all *E. coli* pathotypes (24), which suggests that our MEV immunogen provides good protection against all *E. coli* pathotypes. FimH is present in more than 90% of UPEC strains (83), thereby aligning our infection model.

One of the most important and crucial virulence factors of UPEC is type 1 fimbriae, which mediates bacterial adherence and infection progression (84), and FimH is a well-known tip adhesin part of this fimbriae that mediates the attachment process (85) as well as initiates an inflammatory reaction in the host (86). Therefore, the immunogenicity of our chimeric FimH-targeting vaccine, as a component of the final construct, may reflect reduced bacterial load. However, further *in vitro* studies are needed to test the effectiveness of MEV polyclonal antibodies in inhibiting UPEC isolates from adhering to human bladder cells.

Immunisation after FMT, combined with gut microbiota modulation, was associated with the greatest reduction in bladder UPEC load, approximately 813-fold. To test the underlying mechanisms, we suggest that this vaccine formulation increases NLRP3 levels, thereby promoting the production of antimicrobial peptides that cause bacterial cell lysis, as recently documented (87). Furthermore, FMT-mediated SCFA increases levels, activates the NLRP3 inflammasome, and secretes a network of cytokines that drive cell migration to the site of infection. This interaction has previously been studied (88).

In summary, our designed chimeric vaccine demonstrates strong immunogenicity and elicits both humoral and cellular immune responses against UPEC. As the vaccine includes subunits from proteins conserved amongst all *E. coli* pathotypes, this suggests that our designed vaccine is a candidate that covers all *E. coli* pathotypes. On the other hand, FMT was tested for the first time as a bacterial vaccine adjuvant, and the results clearly show its adjuvanticity. However, the mechanism underlying this adjuvanticity remains incompletely understood; the results suggest that NLRP3 may mediate this effect.

Although our data demonstrate a significant effect of FMT on vaccine-induced immunity, the molecular mechanisms underlying this effect remain to be fully explored. According to the available literature, the adjuvant-like effects of FMT are most likely mediated by microbiota-derived metabolites that activate the NLRP3 inflammasome. However, a major limitation of the present study is the lack of direct microbiome profiling and metabolite measurements, and thus, this mechanism remains largely speculative. Consequently, upcoming studies using 16S rRNA sequencing, metagenomic analysis, and SCFA quantification are vital to conclusively approve the proposed mechanism and identify the specific microbial taxa responsible for this immunomodulatory effect. Another critical factor is validating the success and time stability of the microbiota alterations in recipient mice after transplantation. Therefore, it will be important to characterise gut microbiota shifts and community dynamics post-FMT in future work to better correlate specific microbial changes with improved vaccine efficacy. The present study does not assess the endotoxin content of the candidate vaccine, which may affect the outcomes. Lastly, because of limited facilities for supplying local *E. coli* pathotypes other than UPEC and the number of mice required, part of the hypothesis of the present study has not yet been tested, warranting future work using other *E. coli* pathotypes.

## Data Availability

The datasets presented in this study can be found in online repositories. The names of the repository/repositories and accession number(s) can be found in the article/**Supplementary Material**.
