# Supplementary material for: Chimeric vaccine based on Iraqi HLA alleles against a predominant local Escherichia coli phylogroup
Source: Front Immunol. 2026 Aug 18;17:1875830. doi: 10.3389/fimmu.2026.1875830 (PMC13529670; doi:10.3389/fimmu.2026.1875830)
Supplement: Supplementary file 1 [file Table1.docx]

> PZ267928 Chimeric Vaccine Candidate AZIZMEV against E. coli

ATGGGTATCATCAACACCCTGTGCAAATACTACTGCCGTGTTCGTGGTGGTCGTTGCTGCGTTTGCTCTTGCTGCCCGAAAGAAGAACAGATCGGTAAATGCTCTACCCGTGGTCGTAAATGCTGCCGTCGTAAAAAAGAAGCTGCTAAAGCTAAATTCGTTGCTGCTTGGACCCTGAAAGCTGCTGCTGAAGCTGCTAAATCTTCTGCTGGTGGTGTTGCTATCAAAGGTGGTGGTTCTATGGTTGGTCGTCAGGGTCGTGGTAAAGGTGGTTCTCAGACCCTGGAACCGCGTGCTCAGTACGGTGGTGGTTCTGAACCGGTTCGTACCGTTGACGCTCTGGGTGGTGGTTCTAAACTGGACGAATCTGTTAACCGTGTTGGTGGTGGTTCTACCCTGGAACCGCGTGCTCAGTACCTGGGTGGTGGTTCTAAAACCGACGACTTCACCTTCAACTACGGTGGTGGTTCTAAAGTTACCCTGGACACCGCTACCTACGGTGGTGGTTCTGCTATGTGGCGTTACCTGTACTCTATGGGTGGTGGTTCTTCTGGTGTTCGTGTTGGTGAATCTCTGGGTCCGGGTCCGGGTTCTGACCCGTCTTACTTCAACGACTTCGACAACAAATACGGTTCTGGTCCGGGTCCGGGTGGTGACTACCAGATGGTTGGTCGTCAGGGTCGTGGTAAAGCTGACGGTCCGGGTCCGGGTATGGACGCTACCATCACCCCGCACTACATGCACCGTCGTGGTAACGGTCCGGGTCCGGGTTACGCTCAGCCGTTCAAAAAATACGACGGTGACAAAGCTGAACAGGGTCCGGGTCCGGGTGGTAACCGTTTCTACGTTCGTAAAATCCGTTTCGAAGGTAACGACAAAAAACCGACCGGTGGTTGCGACGCTTCTGCTCGTGACGTTACCGTTACCCTGAAAAAAGACGAACACCCGAACGACGACTCTTCTCGTCGTTGGCTGTTCTACTGGAAAAAAACCCGTTGGGGTTACGGTGACGGTCTGGGTGGTAAAGAAATGCCGTTCCACCACCACCACCACCAC

**Supplementary Data Sheet 1:** The official text-based FASTA sequence of the optimised codons for the predicted epitopes by the Jcat online tool. Amino acids sequence (*n*= 352) was reverse translated and optimised for heterogenous expression in BL21 *E. coli*. CAI and GC content adjusted to 1 and 53.598% to exclude rare codons.

**Supplementary Figure 1:** WebLogo representation of conservation across the predicted FimH epitopes. The sequence conservation and the relative amino acid frequencies were calculated from a multiple sequence alignment of *n* = 80 non-redundant protein sequences from the NCBI database. Alignment was performed with ClustalW/Omega, and the sequence logo was generated with WebLogo 3. The height of each letter is proportional to its frequency at this position in bits. Epitopes in green represent regions with a high sequence conservation threshold. Error bars represent sample variance.

**Supplementary Figure 2:** WebLogo representation of conservation across the predicted LptD epitopes. The sequence conservation and the relative amino acid frequencies were calculated from a multiple sequence alignment of *n* = 50 non-redundant protein sequences from the NCBI database. Alignment was performed with ClustalW/Omega, and the sequence logo was generated with WebLogo 3. The height of each letter is proportional to its frequency at this position in bits. Epitopes in green represent regions with a high sequence conservation threshold. Error bars represent sample variance.

**Supplementary Figure 3:** WebLogo representation of conservation across the predicted BamA epitopes. The sequence conservation and the relative amino acid frequencies were calculated from a multiple sequence alignment of *n* = 67 non-redundant protein sequences from the NCBI database. Alignment was performed with ClustalW/Omega, and the sequence logo was generated with WebLogo 3. The height of each letter is proportional to its frequency at this position in bits. Epitopes in green represent regions with a high sequence conservation threshold. Error bars represent sample variance.

**Supplementary Figure 4:** Primary structure of the candidate vaccine. Yellow letters refer to internal β-defensin adjuvant, Brown letters refer to internal PADRE adjuvant, Red letters refer to CTL epitopes, Blue letters refer to HTL epitopes, Green letters refer to B-cell epitopes, Black letters refer to internal linkers.

**Supplementary Table 1:** Humoral immune responses mediated by different formulations of the chimeric vaccine.

| Immunological parameters | Time | Control | MEV | PLGA | FMT | A/F | P-values |
| --- | --- | --- | --- | --- | --- | --- | --- |
|  | Mean±S.D. | | | | | |  |
| IgG (µg/mL) | day 30 | 1.28±0.16 | 1.59±0.09 | 1.42±0.02 | 1.52±0.11 | 1.67±0.12 | ˂0.0001 |
|  | day75 | 1.38±0.09 | 1.60±0.11 | 1.7±0.09 | 1.67±0.11 | 1.81±0.09 |  |
| IL-4 (pg/mL) | day 30 | 11.32±1.34 | 15.10±1.18 | 12.24±0.64 | 13.2±0.66 | 15.58±0.36 | ˂0.0001 |
|  | day75 | 12.35±1.15 | 12.60±1.08 | 14.75±0.47 | 13.21±1.07 | 18.24±1.34 |  |
| INF-γ (pg/mL) | day 30 | 18.39±0.78 | 28.98±5.84 | 24.19±1.06 | 26.82±1.24 | 24.69±0.82 | ˂0.0001 |
|  | day75 | 18.75±6.29 | 29.02±5.79 | 30.59±3.21 | 24.09±4.08 | 34.13±3.71 |  |
| NLRP3 (pg/mL) | day 30 | 501.42±28.25 | 566.48±44.77 | 622.36±76.63 | 447.91±25.23 | 709.92±52.41 | 0.0004 |
|  | day75 | 522.81±31.35 | 723.78±49.88 | 673.46±61.31 | 641.86±12.14 | 756.52±54.73 |  |

**MEV = immunisation with purified 100 µg of the chimeric vaccine alone, PLGA = injection of purified 100 µg of the PLGA-encapsulated chimeric protein, FMT = immunisation with 100 µg of the chimeric vaccine after faecal microbiota transplantation, A/F= immunisation with 100 µg of the chimeric vaccine after FMT combined with modulation of the gut microbiota.**

**Supplementary Table 2:** Reduction of bacterial load in the bladder stimulated by different formulations of the chimeric vaccine.

| **Groups** | **Log of bacterial growth** | **Fold reduction** |
| --- | --- | --- |
| **Control** | 9.07±0.53 | 0 |
| **MEV** | 6.51±1.51 | 360 |
| **PLGA** | 6.62±0.47 | 282 |
| **FMT** | 7.88±1.29 | 15.5 |
| **A/F** | 6.16±0.38 | 813 |
| **P-value** | 0.003 | |

- **MEV = immunisation with purified 100 µg of the chimeric vaccine alone, PLGA = injection of purified 100 µg of the PLGA-encapsulated chimeric protein, FMT = immunisation with 100 µg of the chimeric vaccine after faecal microbiota transplantation, A/F= immunisation with 100 µg of the chimeric vaccine after FMT combined with modulation of the gut microbiota.**
- **Fold reduction was in comparison with the control group and calculated using the formula: Folding reduction =** $\boldsymbol{10}^{\boldsymbol{(Log of control-Log of the tested group)}}$
